# Targeted Gene Sanger Sequencing Should Remain the First-Tier Genetic Test for Children Suspected to Have the Five Common X-Linked Inborn Errors of Immunity

**DOI:** 10.3389/fimmu.2022.883446

**Published:** 2022-07-08

**Authors:** Koon-Wing Chan, Chung-Yin Wong, Daniel Leung, Xingtian Yang, Susanna F. S. Fok, Priscilla H. S. Mak, Lei Yao, Wen Ma, Huawei Mao, Xiaodong Zhao, Weiling Liang, Surjit Singh, Mohamed-Ridha Barbouche, Jian-Xin He, Li-Ping Jiang, Woei-Kang Liew, Minh Huong Thi Le, Dina Muktiarti, Fatima Johanna Santos-Ocampo, Reda Djidjik, Brahim Belaid, Intan Hakimah Ismail, Amir Hamzah Abdul Latiff, Way Seah Lee, Tong-Xin Chen, Jinrong Liu, Runming Jin, Xiaochuan Wang, Yin Hsiu Chien, Hsin-Hui Yu, Dinesh Raj, Revathi Raj, Jenifer Vaughan, Michael Urban, Sylvia van den Berg, Brian Eley, Anselm Chi-Wai Lee, Mas Suhaila Isa, Elizabeth Y. Ang, Bee Wah Lee, Allen Eng Juh Yeoh, Lynette P. Shek, Nguyen Ngoc Quynh Le, Van Anh Thi Nguyen, Anh Phan Nguyen Lien, Regina D. Capulong, Joanne Michelle Mallillin, Jose Carlo Miguel M. Villanueva, Karol Anne B. Camonayan, Michelle De Vera, Roxanne J. Casis-Hao, Rommel Crisenio M. Lobo, Ruby Foronda, Vicky Wee Eng Binas, Soraya Boushaki, Nadia Kechout, Gun Phongsamart, Siriporn Wongwaree, Chamnanrua Jiratchaya, Mongkol Lao-Araya, Muthita Trakultivakorn, Narissara Suratannon, Orathai Jirapongsananuruk, Teerapol Chantveerawong, Wasu Kamchaisatian, Lee Lee Chan, Mia Tuang Koh, Ke Juin Wong, Siew Moy Fong, Meow-Keong Thong, Zarina Abdul Latiff, Lokman Mohd Noh, Rajiva de Silva, Zineb Jouhadi, Khulood Al-Saad, Pandiarajan Vignesh, Ankur Kumar Jindal, Amit Rawat, Anju Gupta, Deepti Suri, Jing Yang, Elaine Yuen-Ling Au, Janette Siu-Yin Kwok, Siu-Yuen Chan, Wayland Yuk-Fun Hui, Gilbert T. Chua, Jaime Rosa Duque, Kai-Ning Cheong, Patrick Chun Yin Chong, Marco Hok Kung Ho, Tsz-Leung Lee, Wilfred Hing-Sang Wong, Wanling Yang, Pamela P. Lee, Wenwei Tu, Xi-Qiang Yang, Yu Lung Lau

**Affiliations:** ^1^ Department of Paediatrics and Adolescent Medicine, School of Clinical Medicine, Li Ka Shing Faculty of Medicine, The University of Hong Kong, Hong Kong, Hong Kong SAR, China; ^2^ Department of Immunology, Ministry of Education Key Laboratory of Major Diseases in Children, Beijing Children’s Hospital, Capital Medical University, National Center for Children’s Health, Beijing, China; ^3^ Children’s Hospital, Chongqing Medical University, Chongqing, China; ^4^ Shenzhen Primary Immunodeficiency Diagnostic and Therapeutic Laboratory, The University of Hong Kong-Shenzhen Hospital, Shenzhen, China; ^5^ Pediatric Allergy and Immunology Unit, Department of Pediatrics, Advanced Pediatrics Centre, Postgraduate Institute of Medical Education and Research, Chandigarh, India; ^6^ Institut Pasteur de Tunis, Université Tunis El Manar, Tunis, Tunisia; ^7^ Department of Respiratory Medicine, Beijing Children’s Hospital, Capital Medical University, National Center for Children’s Health, Beijing, China; ^8^ Department of Paediatric Medicine, KK Women’s and Children’s Hospital, Singapore, Singapore; ^9^ Department of Immuno-Allergology and Rheumatology, National Hospital of Paediatrics, Hanoi, Vietnam; ^10^ Department of Child Health, Faculty of Medicine Universitas Indonesia-Cipto Mangunkusumo Hospital, Jakarta, Indonesia; ^11^ Section of Allergy and Immunology, Department of Pediatrics, Makati Medical Center, Makati City, Philippines; ^12^ Department of Medical Immunology, Beni Messous University Hospital Centre, University of Algiers 1, Algiers, Algeria; ^13^ Clinical Immunology Unit, Department of Paediatrics, Faculty of Medicine and Health Sciences, Universiti Putra Malaysia, Selangor, Malaysia; ^14^ Allergy and Immunology Centre, Pantai Hospital Kuala Lumpur, Kuala Lumpur, Malaysia; ^15^ Department of Paediatrics, Faculty of Medicine, University Malaya, Kuala Lumpur, Malaysia; ^16^ Shanghai Children’s Medical Center, School of Medicine, Shanghai Jiao Tong University, Shanghai, China; ^17^ Department of Pediatrics, Union Hospital, Tongji Medical College, Huazhong University of Science and Technology, Wuhan, China; ^18^ Department of Clinical Immunology, Children’s Hospital of Fudan University, Shanghai, China; ^19^ Department of Medical Genetics and Pediatrics, National Taiwan University Hospital, Taipei, Taiwan; ^20^ Division of Allergy, Immunology and Rheumatology, Department of Pediatrics, National Taiwan University Children’s Hospital, Taipei, Taiwan; ^21^ Department of Paediatrics, Holy Family Hospital, University of Delhi, New Delhi, India; ^22^ Department of Paediatric Haematology, Oncology, Blood and Marrow Transplantation, Apollo Hospitals, Chennai, India; ^23^ Department of Molecular Medicine and Haematology, National Health Laboratory Services, University of the Witwatersrand, Johannesburg, South Africa; ^24^ Division of Molecular Biology and Human Genetics, University of Stellenbosch Western Cape, Pretoria, South Africa; ^25^ Department of Immunology, Ampath and Department of Paediatrics and Child Health, University of Pretoria and Steve Biko Academic Hospital, Pretoria, South Africa; ^26^ Department of Paediatrics and Child Health, University of Cape Town and Red Cross War Memorial Children’s Hospital, Cape Town, South Africa; ^27^ Children’s Haematology and Cancer Center, Mount Elizabeth Hospital, Singapore, Singapore; ^28^ Khoo Teck Puat-National University Children’s Medical Institute, National University Health System, Singapore, Singapore; ^29^ Department of Paediatrics, Yong Loo Lin School of Medicine, National University of Singapore, Singapore, Singapore; ^30^ Singapore Institute for Clinical Sciences, Agency for Science, Technology and Research (A*STAR), Singapore, Singapore; ^31^ National Hospital of Pediatrics, Hanoi, Vietnam; ^32^ Department of Rheumatology, Allergy, and Immunology, Vietnam National Children's Hospital, Hanoi, Vietnam; ^33^ Children’s Hospital 1, Ho Chi Minh City, Vietnam; ^34^ Department of Pediatrics, ManilaMed, Manila, Philippines; ^35^ Child and Adult Allergy, Asthma and Immunology General Emilio Aguinaldo Memorial Hospital, Cavite, Philippines; ^36^ Section of Allergy and Clinical Immunology, Department of Pediatrics, University of Santo Tomas Hospital, Manila, Philippines; ^37^ Philippine General Hospital, University of the Philippines, Manila, Philippines; ^38^ Section of Allergy and Immunology, The Medical City, Pasig, Philippines; ^39^ Division of Allergy and Clinical Immunology, Department of Pediatrics, Philippine General Hospital, Manila, Philippines; ^40^ Section of Allergy Asthma and Immunology, Fe del Mundo Medical Center, Quezon City, Philippines; ^41^ Department of Pediatrics, University of the East Ramon Magsaysay Memorial Medical Center, Quezon City, Philippines; ^42^ De La Salle Health Sciences Institute, Dasmarinas, Philippines; ^43^ Unit of Genetics, Laboratory of Molecular and Cellular Biology, Faculty of Biological Sciences, University of Sciences and Technology “HouariBoumediene”, Algiers, Algeria; ^44^ Department of Immunology, Pasteur Institute of Algeria/Faculty of Medicine, Algiers, Algeria; ^45^ Department of Pediatrics, Queen Sirikit National Institute of Child Health, Bangkok, Thailand; ^46^ Department of Pediatrics, Faculty of Medicine, Chiang Mai University, Chiang Mai, Thailand; ^47^ Center of Excellence for Allergy and Clinical Immunology, Division of Allergy, Immunology and Rheumatology, Department of Pediatrics, King Chulalongkorn Memorial Hospital, the Thai Red Cross Society, Faculty of Medicine, Chulalongkorn University, Bangkok, Thailand; ^48^ Division of Allergy and Clinical Immunology, Department of Pediatrics, Faculty of Medicine Siriraj Hospital, Mahidol University, Bangkok, Thailand; ^49^ Division of Allergy and Clinical Immunology, Department of Medicine, Phramongkutklao Hospital, Bangkok, Thailand; ^50^ Division of Pediatrics Allergy and Immunology, Department of Pediatrics, Faculty of Medicine Ramathibodi Hospital, Mahidol University, Bangkok, Thailand; ^51^ Subang Jaya Medical Centre, Subang Jaya, Malaysia; ^52^ Department of Paediatrics, Likas Hospital, Ministry of Health, Sabah, Malaysia; ^53^ Genetics and Metabolism Unit, Department of Paediatrics, Faculty of Medicine, University of Malaya, Kuala Lumpur, Malaysia; ^54^ Department of Pediatrics, Universiti Kebangsaan Malaysia, Kuala Lumpur, Malaysia; ^55^ Department of Paediatrics, Hospital Tunku Azizah, Ministry of Health Malaysia, Kuala Lumpur, Malaysia; ^56^ Department of Immunology, Medical Research Institute, Colombo, Sri Lanka; ^57^ Department of Pediatric Infectious Diseases, Children’s Hospital CHU Ibn Rochd, University Hassan 2, Casablanca, Morocco; ^58^ Department of Pediatrics, Salmaniya Medical Complex, Manama, Bahrain; ^59^ Division of Clinical Immunology, Department of Pathology, Queen Mary Hospital, Hong Kong, Hong Kong SAR, China; ^60^ Division of Transplantation and Immunogenetics, Department of Pathology, Queen Mary Hospital, Hong Kong, Hong Kong SAR, China; ^61^ Hong Kong Children’s Hospital, Hong Kong, Hong Kong SAR, China; ^62^ Virtus Medical, Hong Kong, Hong Kong SAR, China

**Keywords:** inborn errors of immunity, primary immunodeficiency diseases, targeted gene, Sanger sequencing, whole exome sequencing, next generation sequencing

## Abstract

To address inborn errors of immunity (IEI) which were underdiagnosed in resource-limited regions, our centre developed and offered free genetic testing for the most common IEI by Sanger sequencing (SS) since 2001. With the establishment of The Asian Primary Immunodeficiency (APID) Network in 2009, the awareness and definitive diagnosis of IEI were further improved with collaboration among centres caring for IEI patients from East and Southeast Asia. We also started to use whole exome sequencing (WES) for undiagnosed cases and further extended our collaboration with centres from South Asia and Africa. With the increased use of Next Generation Sequencing (NGS), we have shifted our diagnostic practice from SS to WES. However, SS was still one of the key diagnostic tools for IEI for the past two decades. Our centre has performed 2,024 IEI SS genetic tests, with in-house protocol designed specifically for 84 genes, in 1,376 patients with 744 identified to have disease-causing mutations (54.1%). The high diagnostic rate after just one round of targeted gene SS for each of the 5 common IEI (X-linked agammaglobulinemia (XLA) 77.4%, Wiskott–Aldrich syndrome (WAS) 69.2%, X-linked chronic granulomatous disease (XCGD) 59.5%, X-linked severe combined immunodeficiency (XSCID) 51.1%, and X-linked hyper-IgM syndrome (HIGM1) 58.1%) demonstrated targeted gene SS should remain the first-tier genetic test for the 5 common X-linked IEI.

## Introduction

Inborn errors of immunity (IEI), previously known as primary immunodeficiency diseases (PIDD), arise from intrinsic defects in immunity, with most due to genetic mutations, and comprise over 400 diseases that could present with a diverse range of disorders including infection, autoimmunity, inflammation, malignancy, and allergy (1, 2). These multitudes of disorders could present with a wide spectrum of phenotypes of varying severities, resulting in difficulty recognising and diagnosing IEI promptly and accurately, especially in resource-limited countries and regions (3).

With rapid advance in both immunological and genetic studies in IEI including newborn screening for severe combined immunodeficiency (SCID) over the last 20 years, the prognosis of patients with IEI living in resource-rich countries and regions have improved enormously due to rapid and accurate genetic diagnosis with treatment tailored to specific IEI, together with family counseling regarding recurrence risk and reproductive choices (3–5). However, for most countries and regions of Asia and Africa, many patients with suspected IEI now still do not have ready access to these diagnostic and therapeutic approaches, let alone 20 years ago, resulting in underdiagnosis of IEI and a protracted diagnostic odyssey for many families (6).

To improve awareness and recognition of IEI in our region, we started to offer e-consultation and genetic investigations free of charge for patients suspected to have IEI referred to us by our collaborators since 2001. This was built on our paediatric immunology service started in 1988, with us having rapidly acquired the in-house capacity to diagnose IEI genetically and treat the more common IEI effectively (7–17). With more experience, we started to offer the research based targeted gene Sanger sequencing (SS) for the 5 common X-linked IEI, namely X-linked agammaglobulinemia (XLA), Wiskott-Aldrich syndrome (WAS), X-linked chronic granulomatous disease (XCGD), X-linked hyper-IgM (HIGM1) and X-linked severe combined immunodeficiency (XSCID), to our collaborators in South-East Asia and mainland China initially, followed by those in South Asia and Africa. The collaboration has resulted in providing accurate genetic diagnosis leading to appropriate management of these patients as well as increasing awareness of IEI in these countries and regions (18–31).

Over the years, we have increased the number of targeted genes subjected to SS to more than 80, as well as helped our collaborators in setting up their local genetic diagnostic service through sharing of protocols and primers, resulting in local centres with expertise and diagnostics for IEI without the need to refer patients with suspected IEI to us for genetic diagnosis (32–42).

Since 2009, we started to use next generation sequencing (NGS) to investigate patients with suspected IEI whose genetic mutations could not be identified by targeted gene SS. In the same year, we established the Asian Primary Immunodeficiency (APID) Network to provide an electronic platform for both data management and better consultative service for our collaborators (43, 44).

In this study, we aimed to review the role of targeted gene SS in the diagnostic pathway for patients with suspected IEI referred to us from 2001 to 2021, to define which suspected IEI should be subjected to targeted gene SS before offering NGS, with criteria that the gene is the most commonly found to be causal among all the genes that are associated with that clinical phenotype, and with at least a 50% diagnostic rate using one round of SS.

## Materials and Methods

### Patients

Patients with suspected IEI referred to us from different centres over a 20-year period (2001–2021) were included. Various diagnostic work up including laboratory tests and immunological assays were done in the referring centres. Referring clinicians would send us the clinical details and laboratory findings, which would be deposited in our APID network database. Only those patients with clinical presentation indicative of IEI would be followed up (currently can refer to the IUIS phenotypic classification) (2). Cases with HIV infection or other known causes of immune compromise would be excluded. One or several rounds of e-consultation would be conducted between the referring clinicians and the corresponding author who ultimately decided on which targeted gene SS would be done, with clinical and laboratory criteria specific to each top X-linked gene applied listed here below. X-linked genes would be normally sequenced in boys born of non-consanguineous marriages with a non-conflicting family history only, e.g., without affected sisters. Onset of recurrent bacterial infections or enteroviral infections approximately after 6 months of age, and if available, very low IgG level and B cell count would prompt the immediate sequencing of the BTK gene. The WAS gene was sequenced in boys with recurrent bacterial, viral, and fungal infections, eczema, and importantly, thrombocytopenia. The CYBB gene would be sequenced in boys with recurrent bacterial and fungal infections, BCGitis or BCGosis, and if available, a positive nitroblue tetrazolium test (NBT) or dihydrorhodamine (DHR) 123 test. The IL2RG gene was sequenced in boys presenting in first few months of life with recurrent severe infections, low absolute lymphocyte count, and if available, a very low T or NK cell count. The CD40LG gene was sequenced in boys with recurrent sinopulmonary infections, liver and biliary tract disease, and if available, a high IgM level accompanied by low IgG and IgA levels. Additional or more advanced laboratory investigations were normally not requested before proceeding to genetic testing as most patients were referred from resource-limited settings. Less than 5% of referral cases were not offered genetic testing due to insufficient clinical details. Once genomic DNA were received, genetic diagnosis by research-based targeted gene SS was then performed by our centre free of charge. The study was approved by the Clinical Research Ethics Review Board of The University of Hong Kong and Queen Mary Hospital (Ref. no. UW 08-301).

### Targeted Gene SS

Genomic DNA was isolated from peripheral blood of patients by different centres, with consent obtained from parents or guardians before blood collection. Polymerase chain reaction (PCR) primer pairs covering entire coding region and flanking splice sites were designed for individual IEI genes. Research-based targeted gene SS was performed by PCR or long PCR direct SS of both sense and antisense strands of DNA as described in our previous studies (19, 20, 22–25). Homology analyses with reference sequences were performed by Basic Local Alignment Search Tool (BLAST). Mutations, identified by bioinformatics analysis, were described with reference to Human Genome Variation Society (HGVS) nomenclature (45). For those patients with typical phenotypes including the 5 common IEI, relevant single targeted gene SS has been offered in the first round of screening, e.g., *BTK*(Bruton tyrosine kinase) gene for XLA, *WAS* (WASP actin nucleation promoting factor) gene for WAS, *CYBB* (cytochrome b-245 beta chain) gene for XCGD, *IL2RG* (interleukin 2 receptor subunit gamma) gene for XSCID and *CD40LG* (CD40 ligand) gene for XHIM. For the other IEI, targeted gene or gene panel SS were offered at the same time. Further targeted gene tests were performed if no causal mutation identified in the previous round of SS.

## Results

**Figure 1:**
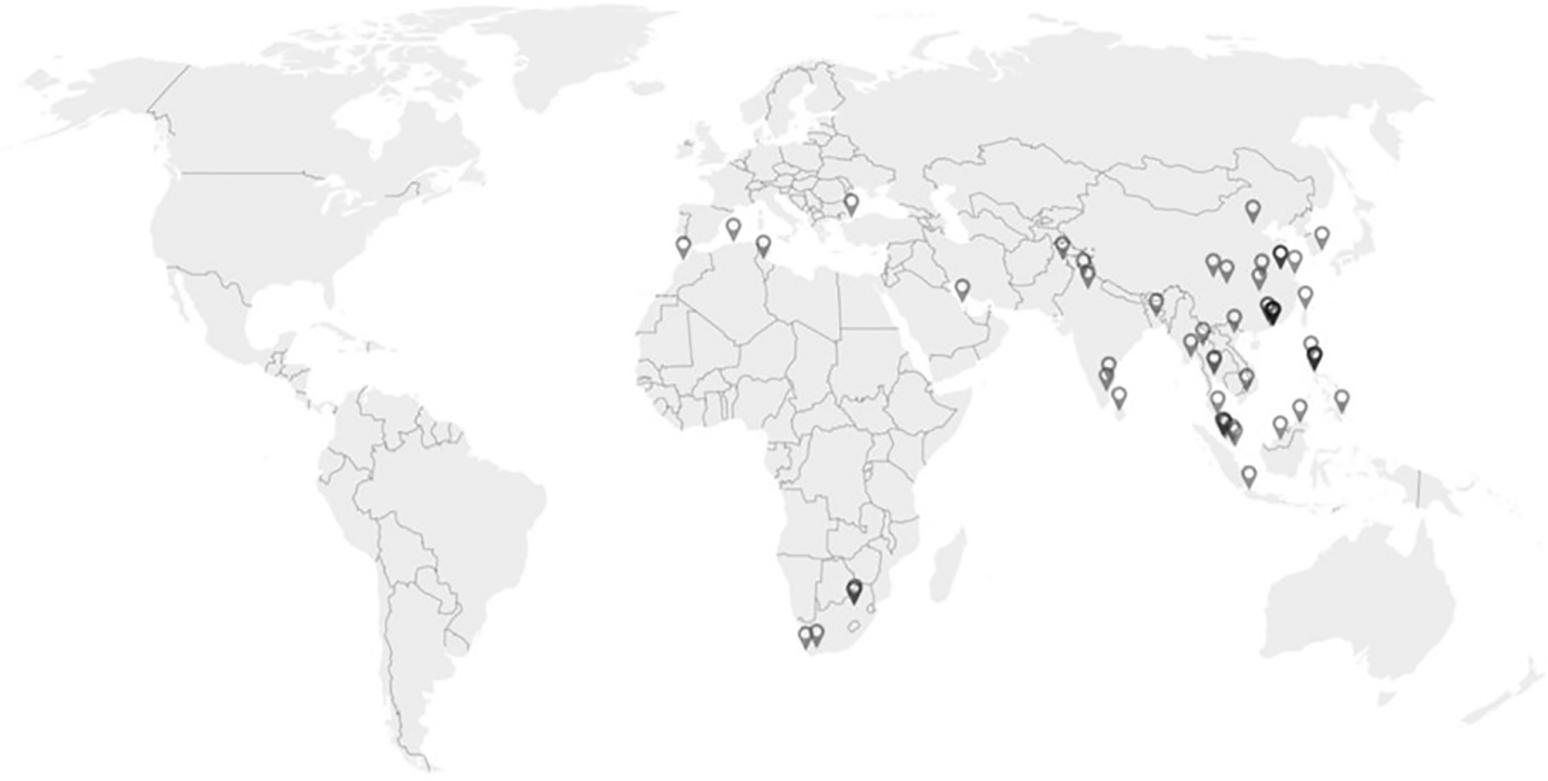
Map showing 72 referring centres in 17 countries. (Created with Datawrapper).

From 2001 to 2021, 1,376 patients with suspected IEI have been referred from different centres as shown in **Figure 1**. We have developed 84 different IEI targeted gene tests according to the diversity of IEI cases referred. Totally, we have performed 2,024 targeted gene SS for all these IEI patients referred, with 744 patients identified to have disease-causing mutations. The positive diagnostic rates among patients and tests are 54.1% (744 out of 1,376 patients) and 36.8% (744 out of 2,024 SS) respectively, with 1.47 SS performed per patient on average. The details of the mutations were described in the **Tables 1**–**4**, and **Supplementary Tables 1, 2**. **Tables 1**–**4**, and **Supplementary Table 1** show all causal mutations found in the corresponding genes of the 5 common IEI while **Supplementary Table 2** for all other IEI genes.

**Table 1:** Causal mutations identified in WAS gene (Reference Sequence LRG_125) of the WAS patients.

Patient ID	Gene	Mutant allele	cDNA/nucleotide change	Protein change	Mutant type
WAS-016A	*WAS*	X-linked	LRG_125t1:c.35G>C LRG_125t1:c.62del	G12A N21Tfs*24	Missense Frameshift
WAS-051A	*WAS*	X-linked	LRG_125t1:c.58C>T	Q20X	Missense
WAS-149A	*WAS*	X-linked	LRG_125t1:c.91G>A	E31K	Missense
WAS-039A	*WAS*	X-linked	LRG_125t1:c.116T>G	L39R	Missense
WAS-083A	*WAS*	X-linked	**LRG_125t1:c.134C>T**	**T45M**	Missense
WAS-102A	*WAS*	X-linked	**LRG_125t1:c.134C>T**	**T45M**	Missense
WAS-088A	*WAS*	X-linked	LRG_125t1:c.167C>T	A56V	Missense
WAS-056A	*WAS*	X-linked	LRG_125t1:c.190T>A	W64R	Missense
WAS-025A	*WAS*	X-linked	LRG_125t1:c.217T>C	C73R	Missense
WAS-045A	*WAS*	X-linked	LRG_125t1:c.218G>A	C73Y	Missense
WAS-055A	*WAS*	X-linked	LRG_125t1:c.223G>A	V75M	Missense
WAS-048A	*WAS*	X-linked	LRG_125t1:c.245C>A	S82Y	Missense
WAS-121A	*WAS*	X-linked	LRG_125t1:c.256C>T	R86C	Missense
WAS-030A	*WAS*	X-linked	**LRG_125t1:c.257G>A**	**R86H**	Missense
WAS-082A	*WAS*	X-linked	**LRG_125t1:c.257G>A**	**R86H**	Missense
WAS-101A	*WAS*	X-linked	**LRG_125t1:c.257G>A**	**R86H**	Missense
WAS-137A	*WAS*	X-linked	**LRG_125t1:c.257G>A**	**R86H**	Missense
WAS-148A	*WAS*	X-linked	**LRG_125t1:c.257G>A**	**R86H**	Missense
WAS-044A	*WAS*	X-linked	LRG_125t1:c.257G>T	R86L	Missense
WAS-097A	*WAS*	X-linked	LRG_125t1:c.300G>C	E100D	Missense
WAS-070A	*WAS*	X-linked	**LRG_125t1:c.397G>A**	**E133K**	Missense
WAS-131A	*WAS*	X-linked	**LRG_125t1:c.397G>A**	**E133K**	Missense
WAS-136A	*WAS*	X-linked	**LRG_125t1:c.397G>A**	**E133K**	Missense
WAS-151A	*WAS*	X-linked	**LRG_125t1:c.397G>A**	**E133K**	Missense
WAS-001A	*WAS*	X-linked	LRG_125t1:c.1354G>T	E452X	Missense
WAS-049A	*WAS*	X-linked	LRG_125t1:c.1376C>T LRG_125t1:c.1421T>A	P459L M474K	Missense Missense
WAS-071A	*WAS*	X-linked	LRG_125t1:c.1378C>T	P460S	Missense
WAS-154A	*WAS*	X-linked	LRG_125t1:c.97C>T	Q33*	Nonsense
WAS-110A	*WAS*	X-linked	**LRG_125t1:c.100C>T**	R34*	Nonsense
WAS-152A	*WAS*	X-linked	**LRG_125t1:c.100C>T**	R34*	Nonsense
WAS-160A	*WAS*	X-linked	**LRG_125t1:c.100C>T**	R34*	Nonsense
WAS-123A	*WAS*	X-linked	LRG_125t1:c.107_108del	F36*	Nonsense
WAS-029A	*WAS*	X-linked	**LRG_125t1:c.121C>T**	R41*	Nonsense
WAS-078A	*WAS*	X-linked	**LRG_125t1:c.121C>T**	R41*	Nonsense
WAS-112A	*WAS*	X-linked	**LRG_125t1:c.121C>T**	R41*	Nonsense
WAS-128A	*WAS*	X-linked	LRG_125t1:c.184G>T	E62*	Nonsense
WAS-050A	*WAS*	X-linked	LRG_125t1:c.290G>A	W97*	Nonsense
WAS-100A	*WAS*	X-linked	LRG_125t1:c.100C>T	R34*	Nonsense
WAS-119A	*WAS*	X-linked	LRG_125t1:c.306C>G	Y102*	Nonsense
WAS-158A	*WAS*	X-linked	LRG_125t1:c.403C>T	Q135*	Nonsense
WAS-106A	*WAS*	X-linked	LRG_125t1:c.454C>T	Q152*	Nonsense
WAS-006A	*WAS*	X-linked	LRG_125t1:c.472C>T	Q158*	Nonsense
WAS-023A	*WAS*	X-linked	**LRG_125t1:c.631C>T**	R211*	Nonsense
WAS-028A	*WAS*	X-linked	**LRG_125t1:c.631C>T**	R211*	Nonsense
WAS-033A	*WAS*	X-linked	**LRG_125t1:c.631C>T**	R211*	Nonsense
WAS-087A	*WAS*	X-linked	**LRG_125t1:c.631C>T**	R211*	Nonsense
WAS-107A	*WAS*	X-linked	**LRG_125t1:c.631C>T**	R211*	Nonsense
WAS-124A	*WAS*	X-linked	**LRG_125t1:c.631C>T**	R211*	Nonsense
WAS-126A	*WAS*	X-linked	**LRG_125t1:c.631C>T**	R211*	Nonsense
WAS-127A	*WAS*	X-linked	**LRG_125t1:c.631C>T**	R211*	Nonsense
WAS-018A	*WAS*	X-linked	LRG_125t1:c.995dup	N335*	Nonsense
WAS-117A	*WAS*	X-linked	LRG_125t1:c.1317_1318delinsTT	Q440*	Nonsense
WAS-138A	*WAS*	X-linked	LRG_125t1:c.1336A>T	K446*	Nonsense
WAS-125A	*WAS*	X-linked	LRG_125t1:c.330dup	T111Hfs*11	Frameshift
WAS-004A	*WAS*	X-linked	LRG_125t1:c.350del	F117Sfs*10	Frameshift
WAS-034A	*WAS*	X-linked	LRG_125t1:c.410_419del	F137Sfs*121	Frameshift
WAS-155A	*WAS*	X-linked	LRG_125t1:c.431_432insT	K144Nfs*25	Frameshift
WAS-032A	*WAS*	X-linked	LRG_125t1:c.436del	Q146Kfs*115	Frameshift
WAS-072A	*WAS*	X-linked	LRG_125t1:c.442dup	R148Kfs*21	Frameshift
WAS-094A	*WAS*	X-linked	LRG_125t1:c.472_473dup	Q158Hfs*104	Frameshift
WAS-019A	*WAS*	X-linked	LRG_125t1:c.566del	P189Qfs*72	Frameshift
WAS-015A	*WAS*	X-linked	LRG_125t1:c.587_588del	G196Afs*10	Frameshift
WAS-002A	*WAS*	X-linked	**LRG_125t1:c.649_652dup**	P218fs*5	Frameshift
WAS-003A	*WAS*	X-linked	**LRG_125t1:c.649_652dup**	P218fs*5	Frameshift
WAS-021A	*WAS*	X-linked	LRG_125t1:c.647_659dup	P222Tfs*4	Frameshift
WAS-113A	*WAS*	X-linked	LRG_125t1:c.665dup	A223Sfs*2	Frameshift
WAS-027A	*WAS*	X-linked	LRG_125t1:c.735del	K245Nfs*16	Frameshift
WAS-008A	*WAS*	X-linked	LRG_125t1:c.950del	P317Hfs*128	Frameshift
WAS-059A	*WAS*	X-linked	LRG_125t1:c.1001del	G334Vfs*111	Frameshift
WAS-010A	*WAS*	X-linked	LRG_125t1:c.1006_1007del	K336Gfs*158	Frameshift
WAS-058A	*WAS*	X-linked	LRG_125t1:c.1023_1024del	L342Afs*152	Frameshift
WAS-156A	*WAS*	X-linked	LRG_125t1:c.1052dup	P352Tfs*143	Frameshift
WAS-012A	*WAS*	X-linked	LRG_125t1:c.1092del	G366Afs*79	Frameshift
WAS-084A	*WAS*	X-linked	LRG_125t1:c.1143del	P383Lfs*62	Frameshift
WAS-141A	*WAS*	X-linked	LRG_125t1:c.1190del LRG_125t1:c.1188_1199del	P397Rfs*48 P401_P404del	Frameshift In-frame Deletion/Insertion
WAS-057A	*WAS*	X-linked	LRG_125t1:c.1219_1235dup	P413Gfs*38	Frameshift
WAS-007A	*WAS*	X-linked	LRG_125t1:c.1265_1275del	A422Gfs*69	Frameshift
WAS-118A	*WAS*	X-linked	LRG_125t1:c.1271dup	L425Pfs70	Frameshift
WAS-099A	*WAS*	X-linked	LRG_125t1:c.1295del	G432Efs*13	Frameshift
WAS-011A	*WAS*	X-linked	LRG_125t1:c.120_132+1dup		Splicing
WAS-009A	*WAS*	X-linked	LRG_125t1:c.132+1G>T		Splicing
WAS-075A	*WAS*	X-linked	LRG_125t1:c.133-1G>A		Splicing
WAS-047A	*WAS*	X-linked	LRG_125t1:c.687G>T	G229=	Splicing
WAS-120A	*WAS*	X-linked	LRG_125t1:c.274-2A>C		Splicing
WAS-031A	*WAS*	X-linked	LRG_125t1:c.360+1G>A		Splicing
WAS-129A	*WAS*	X-linked	LRG_125t1:c.360+5G>C		Splicing
WAS-040A	*WAS*	X-linked	LRG_125t1:c.361-7T>G		Splicing
WAS-109A	*WAS*	X-linked	LRG_125t1:c.361-1G>A		Splicing
WAS-096A	*WAS*	X-linked	LRG_125t1:c.559+1G>A		Splicing
WAS-115A	*WAS*	X-linked	LRG_125t1:c.559+2T>C		Splicing
WAS-063A	*WAS*	X-linked	LRG_125t1:c.734+2T>C		Splicing
WAS-020A	*WAS*	X-linked	**LRG_125t1:c.735-1G>A**		Splicing
WAS-024A	*WAS*	X-linked	**LRG_125t1:c.735-1G>A**		Splicing
WAS-150A	*WAS*	X-linked	**LRG_125t1:c.735-1G>A**		Splicing
WAS-054A	*WAS*	X-linked	**LRG_125t1:c.777+1G>A**		Splicing
WAS-114A	*WAS*	X-linked	**LRG_125t1:c.777+1G>A**		Splicing
WAS-134A	*WAS*	X-linked	**LRG_125t1:c.777+1G>A**		Splicing
WAS-133A	*WAS*	X-linked	LRG_125t1:c.777+2dup		Splicing
WAS-061A	*WAS*	X-linked	LRG_125t1:c.777+3G>C		Splicing
WAS-014A	*WAS*	X-linked	**LRG_125t1:c.777+3_777+6del**		Splicing
WAS-130A	*WAS*	X-linked	**LRG_125t1:c.777+3_777+6del**		Splicing
WAS-013A	*WAS*	X-linked	LRG_125t1:c.931+2T>C		Splicing
WAS-104A	*WAS*	X-linked	LRG_125t1:c.1338+1G>A		Splicing
WAS-139A	*WAS*	X-linked	LRG_125t1:c.1338+2T>G		Splicing
WAS-022A	*WAS*	X-linked	LRG_125t1:c.1453+1G>C		Splicing
WAS-111A	*WAS*	X-linked	LRG_125t1:c.1453+2T>A		Splicing
WAS-103A	*WAS*	X-linked	EX1-EX2del LRG_125t1:c.1378C>T	P460S	Gross Deletion Missense
WAS-089A	*WAS*	X-linked	EX1-EX12del		Gross Deletion

Repeated mutations are in bold. WAS, WASP actin nucleation promoting factor; WAS, Wiskott–Aldrich Syndrome. *translation termination (stop) codon.

**Table 2:** Causal mutations identified in CYBB gene (Reference Sequence LRG_53) of the XCGD patients.

Patient ID	Gene	Mutant allele	cDNA/nucleotide change	Protein change	Mutant type
XCGD-110A	*CYBB*	X-linked	LRG_53t1:c.-65C>T		Regulatory
XCGD-072A	*CYBB*	X-linked	LRG_53t1:c.376T>C	C126R	Missense
XCGD-018A	*CYBB*	X-linked	LRG_53t1:c.577T>C	S193P	Missense
XCGD-004A	*CYBB*	X-linked	LRG_53t1:c.613T>A	F205I	Missense
XCGD-044A	*CYBB*	X-linked	LRG_53t1:c.626A>G	H209R	Missense
XCGD-077A	*CYBB*	X-linked	LRG_53t1:c.665A>G	H222R	Missense
XCGD-062A	*CYBB*	X-linked	LRG_53t1:c.911C>G EX11-EX13del	P304R	Missense Gross Deletion
XCGD-067A	*CYBB*	X-linked	LRG_53t1:c.925G>A	E309K	Missense
XCGD-013A	*CYBB*	X-linked	LRG_53t1:c.935T>A	M312K	Missense
XCGD-145A	*CYBB*	X-linked	LRG_53t1:c.985T>C	C329R	Missense
XCGD-058A	*CYBB*	X-linked	LRG_53t1:c.1014C>A	H338Q	Missense
XCGD-060A	*CYBB*	X-linked	LRG_53t1:c.1016C>A	P339H	Missense
XCGD-111A	*CYBB*	X-linked	LRG_53t1:c.1022C>T	T341I	Missense
XCGD-008A	*CYBB*	X-linked	LRG_53t1:c.1025T>A	L342Q	Missense
XCGD-125A	*CYBB*	X-linked	LRG_53t1:c.1075G>A	G359R	Missense
XCGD-121A	*CYBB*	X-linked	LRG_53t1:c.1154T>G	I385R	Missense
XCGD-038A	*CYBB*	X-linked	LRG_53t1:c.1234G>A	G412R	Missense
XCGD-078A	*CYBB*	X-linked	LRG_53t1:c.1244C>T	P415L	Missense
XCGD-005A	*CYBB*	X-linked	LRG_53t1:c.1498G>C	D500H	Missense
XCGD-136A	*CYBB*	X-linked	LRG_53t1:c.1546T>C	W516R	Missense
XCGD-103A	*CYBB*	X-linked	LRG_53t1:c.1548G>C	W516C	Missense
XCGD-043A	*CYBB*	X-linked	LRG_53t1:c.1583C>G	P528R	Missense
XCGD-120A	*CYBB*	X-linked	LRG_53t1:c.84G>A	W28*	Nonsense
XCGD-106A	*CYBB*	X-linked	LRG_53t1:c.123C>G	Y41*	Nonsense
XCGD-128A	*CYBB*	X-linked	LRG_53t1:c.217C>T	R73*	Nonsense
XCGD-095A	*CYBB*	X-linked	LRG_53t1:c.271C>T	R91*	Nonsense
XCGD-142A	*CYBB*	X-linked	LRG_53t1:c.388C>T	R130*	Nonsense
XCGD-029A	*CYBB*	X-linked	LRG_53t1:c.469C>T	R157*	Nonsense
XCGD-074A	*CYBB*	X-linked	**LRG_53t1:c.469C>T**	R157*	Nonsense
XCGD-101A	*CYBB*	X-linked	**LRG_53t1:c.469C>T**	R157*	Nonsense
XCGD-032A	*CYBB*	X-linked	**LRG_53t1:c.676C>T**	R226*	Nonsense
XCGD-076A	*CYBB*	X-linked	**LRG_53t1:c.676C>T**	R226*	Nonsense
XCGD-107A	*CYBB*	X-linked	**LRG_53t1:c.676C>T**	R226*	Nonsense
XCGD-137A	*CYBB*	X-linked	**LRG_53t1:c.676C>T**	R226*	Nonsense
XCGD-138A	*CYBB*	X-linked	**LRG_53t1:c.676C>T**	R226*	Nonsense
XCGD-019A	*CYBB*	X-linked	**LRG_53t1:c.868C>T**	R290*	Nonsense
XCGD-084A	*CYBB*	X-linked	**LRG_53t1:c.868C>T**	R290*	Nonsense
XCGD-108A	*CYBB*	X-linked	**LRG_53t1:c.868C>T**	R290*	Nonsense
XCGD-147A	*CYBB*	X-linked	**LRG_53t1:c.868C>T**	R290*	Nonsense
XCGD-080A	*CYBB*	X-linked	LRG_53t1:c.1328G>A	W443*	Nonsense
XCGD-059A	*CYBB*	X-linked	LRG_53t1:c.1399G>T	E467*	Nonsense
XCGD-014A	*CYBB*	X-linked	LRG_53t1:c.1437C>A	Y479*	Nonsense
XCGD-006A	*CYBB*	X-linked	LRG_53t1:c.1555G>T	E519*	Nonsense
XCGD-028A	*CYBB*	X-linked	LRG_53t1:c.77_78del	F26Cfs*8	Frameshift
XCGD-083A	*CYBB*	X-linked	LRG_53t1:c.126_130delinsTTTC	R43Ffs*18	Frameshift
XCGD-009A	*CYBB*	X-linked	LRG_53t1:c.713del	V238Gfs*4	Frameshift
XCGD-118A	*CYBB*	X-linked	LRG_53t1:c.714_715insTA	H239Yfs*4	Frameshift
XCGD-139A	*CYBB*	X-linked	LRG_53t1:c.722_726delTAACA	I241fs*243	Frameshift
XCGD-115A	*CYBB*	X-linked	LRG_53t1:c.725_726del	T242Sfs*3	Frameshift
XCGD-037A	*CYBB*	X-linked	LRG_53t1:c.742del	I248Sfs*7	Frameshift
XCGD-003A	*CYBB*	X-linked	**LRG_53t1:c.742dup**	I248Nfs*36	Frameshift
XCGD-102A	*CYBB*	X-linked	**LRG_53t1:c.742dup**	I248Nfs*36	Frameshift
XCGD-113A	*CYBB*	X-linked	**LRG_53t1:c.742dup**	I248Nfs*36	Frameshift
XCGD-030A	*CYBB*	X-linked	LRG_53t1:c.857_867del	V286Afs*58	Frameshift
XCGD-092A	*CYBB*	X-linked	LRG_53t1:c.1038del	E347Rfs*39	Frameshift
XCGD-079A	*CYBB*	X-linked	LRG_53t1:c.1313del	K438Rfs*64	Frameshift
XCGD-010A	*CYBB*	X-linked	LRG_53t1:c.1327del	W443Gfs*59	Frameshift
XCGD-073A	*CYBB*	X-linked	LRG_53t1:c.1332del	C445Afs*57	Frameshift
XCGD-126A	*CYBB*	X-linked	LRG_53t1:c.1565del	T522Kfs*11	Frameshift
XCGD-134A	*CYBB*	X-linked	LRG_53t1:c.1599_1602del	V534Sfs*12	Frameshift
XCGD-090A	*CYBB*	X-linked	LRG_53t1:c.1619_1626dup	A543Kfs*7	Frameshift
XCGD-075A	*CYBB*	X-linked	LRG_53t1:c.70_72del	F24del	In-frame Deletion/Insertion
XCGD-007A	*CYBB*	X-linked	LRG_53t1:c.646_648del	F216del	In-frame Deletion/Insertion
XCGD-048A	*CYBB*	X-linked	LRG_53t1:c.1164_1166delinsATC	388_389delinsES	In-frame Deletion/Insertion
XCGD-129A	*CYBB*	X-linked	LRG_53t1:c.1322_1324del	F441del	In-frame Deletion/Insertion
XCGD-045A	*CYBB*	X-linked	**LRG_53t1:c.45+1G>A**		Splicing
XCGD-100A	*CYBB*	X-linked	**LRG_53t1:c.45+1G>A**		Splicing
XCGD-119A	*CYBB*	X-linked	LRG_53t1:c.45+1G>C		Splicing
XCGD-143A	*CYBB*	X-linked	LRG_53t1:c.45+2delT		Splicing
XCGD-017A	*CYBB*	X-linked	LRG_53t1:c.46-1G>C		Splicing
XCGD-132A	*CYBB*	X-linked	LRG_53t1:c.141+1_141+2del		Splicing
XCGD-093A	*CYBB*	X-linked	LRG_53t1:c.141+3A>T		Splicing
XCGD-001A	*CYBB*	X-linked	**LRG_53t1:c.252G>A**	**A84=**	Splicing
XCGD-002A	*CYBB*	X-linked	**LRG_53t1:c.252G>A**	**A84=**	Splicing
XCGD-104A	*CYBB*	X-linked	**LRG_53t1:c.252G>A**	**A84=**	Splicing
XCGD-114A	*CYBB*	X-linked	**LRG_53t1:c.252G>A**	**A84=**	Splicing
XCGD-015A	*CYBB*	X-linked	LRG_53t1:c.253-1G>A		Splicing
XCGD-089A	*CYBB*	X-linked	LRG_53t1:c.674+6T>C		Splicing
XCGD-109A	*CYBB*	X-linked	LRG_53t1:c.675-1G>T		Splicing
XCGD-042A	*CYBB*	X-linked	LRG_53t1:c.804+2T>C		Splicing
XCGD-071A	*CYBB*	X-linked	LRG_53t1:c.1150_1151+2delAAGT		Splicing
XCGD-098A	*CYBB*	X-linked	LRG_53t1:c.1151+1G>A		Splicing
XCGD-099A	*CYBB*	X-linked	LRG_53t1:c.1314+2T>G		Splicing
XCGD-023A	*CYBB*	X-linked	LRG_53t1:c.1315-2A>C		Splicing
XCGD-061A	*CYBB*	X-linked	EX1-EX13del		Gross Deletion
XCGD-041A	*CYBB*	X-linked	EX7-EX11del		Gross Deletion
XCGD-116A	*CYBB*	X-linked	EX8-EX13del		Gross Deletion
XCGD-026A	*CYBB*	X-linked	LRG_53t1:c.1713A>T	*571Yext*8	Extension

Repeated mutations are in bold. CYBB, cytochrome b-245 beta chain; XCGD, X-linked chronic granulomatous disease. *translation termination (stop) codon.

**Table 3:** Causal mutations identified in IL2RG gene (Reference Sequence LRG_150) of the XSCID patients.

Patient ID	Gene	Mutant allele	cDNA/nucleotide change	Protein change	Mutant type
IL2RG-062A	*IL2RG*	X-linked	LRG_150t1:c.3G>T	M1I	Start Lost
IL2RG-043A	*IL2RG*	X-linked	**LRG_150t1:c.202G>A**	**E68K**	Missense
IL2RG-089A	*IL2RG*	X-linked	**LRG_150t1:c.202G>A**	**E68K**	Missense
IL2RG-080A	*IL2RG*	X-linked	LRG_150t1:c.252C>A	N84K	Missense
IL2RG-142A	*IL2RG*	X-linked	LRG_150t1:c.272A>G	Y91C	Missense
IL2RG-063A	*IL2RG*	X-linked	LRG_150t1:c.304T>C	C102R	Missense
IL2RG-048A	*IL2RG*	X-linked	LRG_150t1:c.340G>T	G114C	Missense
IL2RG-027A	*IL2RG*	X-linked	LRG_150t1:c.365T>C	I122T	Missense
IL2RG-005A	*IL2RG*	X-linked	LRG_150t1:c.371T>C	L124P	Missense
IL2RG-064A	*IL2RG*	X-linked	LRG_150t1:c.383T>C	F128S	Missense
IL2RG-111A	*IL2RG*	X-linked	LRG_150t1:c.386T>A	V129D	Missense
IL2RG-049A	*IL2RG*	X-linked	LRG_150t1:c.618T>A	H206Q	Missense
IL2RG-008A	*IL2RG*	X-linked	**LRG_150t1:c.670C>T**	**R224W**	Missense
IL2RG-047A	*IL2RG*	X-linked	**LRG_150t1:c.670C>T**	**R224W**	Missense
IL2RG-112A	*IL2RG*	X-linked	LRG_150t1:c.675C>A	S225R	Missense
IL2RG-041A	*IL2RG*	X-linked	**LRG_150t1:c.676C>T**	**R226C**	Missense
IL2RG-123A	*IL2RG*	X-linked	**LRG_150t1:c.676C>T**	**R226C**	Missense
IL2RG-004A	*IL2RG*	X-linked	LRG_150t1:c.677G>A	R226H	Missense
IL2RG-115A	*IL2RG*	X-linked	LRG_150t1:c.694G>C	G232R	Missense
IL2RG-079A	*IL2RG*	X-linked	LRG_150t1:c.709T>C	W237R	Missense
IL2RG-015A	*IL2RG*	X-linked	LRG_150t1:c.722G>T	S241I	Missense
IL2RG-009A	*IL2RG*	X-linked	**LRG_150t1:c.854G>A**	**R285Q**	Missense
IL2RG-014A	*IL2RG*	X-linked	**LRG_150t1:c.854G>A**	**R285Q**	Missense
IL2RG-020A	*IL2RG*	X-linked	**LRG_150t1:c.854G>A**	**R285Q**	Missense
IL2RG-022A	*IL2RG*	X-linked	**LRG_150t1:c.854G>A**	**R285Q**	Missense
IL2RG-025A	*IL2RG*	X-linked	**LRG_150t1:c.854G>A**	**R285Q**	Missense
IL2RG-061A	*IL2RG*	X-linked	**LRG_150t1:c.854G>A**	**R285Q**	Missense
IL2RG-083A	*IL2RG*	X-linked	LRG_150t1:c.854G>T	R285L	Missense
IL2RG-076A	*IL2RG*	X-linked	LRG_150t1:c.979_980delinsTT	E327L	Missense
IL2RG-122A	*IL2RG*	X-linked	LRG_150t1:c.979G>A	E327K	Missense
IL2RG-132A	*IL2RG*	X-linked	LRG_150t1:c.184T>A LRG_150t1:c.204G>C	C62S E68D	Missense Missense
IL2RG-147A	*IL2RG*	X-linked	LRG_150t1:c.181C>T	Q61*	Nonsense
IL2RG-067A	*IL2RG*	X-linked	LRG_150t1:c.202G>T	E68*	Nonsense
IL2RG-075A	*IL2RG*	X-linked	LRG_150t1:c.306C>A	C102*	Nonsense
IL2RG-012A	*IL2RG*	X-linked	**LRG_150t1:c.376C>T**	Q126*	Nonsense
IL2RG-103A	*IL2RG*	X-linked	**LRG_150t1:c.376C>T**	Q126*	Nonsense
IL2RG-007A	*IL2RG*	X-linked	LRG_150t1:c.562C>T	Q188*	Nonsense
IL2RG-033A	*IL2RG*	X-linked	LRG_150t1:c.562C>T	Q188*	Nonsense
IL2RG-023A	*IL2RG*	X-linked	LRG_150t1:c.711G>A	W237*	Nonsense
IL2RG-096A	*IL2RG*	X-linked	LRG_150t1:c.811G>T	G271*	Nonsense
IL2RG-098A	*IL2RG*	X-linked	**LRG_150t1:c.865C>T**	R289*	Nonsense
IL2RG-141A	*IL2RG*	X-linked	**LRG_150t1:c.865C>T**	R289*	Nonsense
IL2RG-146A	*IL2RG*	X-linked	**LRG_150t1:c.865C>T**	R289*	Nonsense
IL2RG-104A	*IL2RG*	X-linked	LRG_150t1:c.929G>A	W310*	Nonsense
IL2RG-032A	*IL2RG*	X-linked	LRG_150t1:c.982C>T	R328*	Nonsense
IL2RG-028A	*IL2RG*	X-linked	LRG_150t1:c.127del	T43Pfs*28	Frameshift
IL2RG-003A	*IL2RG*	X-linked	LRG_150t1:c.310_311delinsG	H104Afs*43	Frameshift
IL2RG-016A	*IL2RG*	X-linked	LRG_150t1:c.359dup	E121Gfs*47	Frameshift
IL2RG-055A	*IL2RG*	X-linked	**LRG_150t1:c.362del**	E121Gfs*26	Frameshift
IL2RG-088A	*IL2RG*	X-linked	**LRG_150t1:c.362del**	E121Gfs*26	Frameshift
IL2RG-074A	*IL2RG*	X-linked	LRG_150t1:c.406_415del	R136Gfs*8	Frameshift
IL2RG-018A	*IL2RG*	X-linked	LRG_150t1:c.421del	Q141Rfs*6	Frameshift
IL2RG-017A	*IL2RG*	X-linked	**LRG_150t1:c.507del**	Q169Hfs*2	Frameshift
IL2RG-058A	*IL2RG*	X-linked	**LRG_150t1:c.507del**	Q169Hfs*2	Frameshift
IL2RG-120A	*IL2RG*	X-linked	LRG_150t1:c.658_659del	T220Vfs*8	Frameshift
IL2RG-040A	*IL2RG*	X-linked	LRG_150t1:c.741dup	S248Efs*55	Frameshift
IL2RG-097A	*IL2RG*	X-linked	LRG_150t1:c.741del	S248Afs*25	Frameshift
IL2RG-001A	*IL2RG*	X-linked	LRG_150t1:c.835del	V279Cfs*15	Frameshift
IL2RG-002A	*IL2RG*	X-linked	LRG_150t1:c.855-72_925-11del	T286Pfs*57	Frameshift
IL2RG-145A	*IL2RG*	X-linked	LRG_150t1:c.115+1G>A		Splicing
IL2RG-118A	*IL2RG*	X-linked	LRG_150t1:c.115+2T>C		Splicing
IL2RG-143A	*IL2RG*	X-linked	LRG_150t1:c.270-2A>G		Splicing
IL2RG-035A	*IL2RG*	X-linked	**LRG_150t1:c.270-15A>G**		Splicing
IL2RG-059A	*IL2RG*	X-linked	**LRG_150t1:c.270-15A>G**		Splicing
IL2RG-129A	*IL2RG*	X-linked	LRG_150t1:c.455-2A>T		Splicing
IL2RG-144A	*IL2RG*	X-linked	LRG_150t1:c.757_757+1delinsTC		Splicing
IL2RG-113A	*IL2RG*	X-linked	LRG_150t1:c.854+3G>T		Splicing
IL2RG-006A	*IL2RG*	X-linked	**LRG_150t1:c.854+5G>A**		Splicing
IL2RG-011A	*IL2RG*	X-linked	**LRG_150t1:c.854+5G>A**		Splicing
IL2RG-042A	*IL2RG*	X-linked	LRG_150t1:c.855-2A>C		Splicing
IL2RG-121A	*IL2RG*	X-linked	LRG_150t1:c.855-2A>T		Splicing

Repeated mutations are in bold. IL2RG, interleukin 2 receptor subunit gamma; XSCID, X-linked severe combined immunodeficiency. *translation termination (stop) codon.

**Table 4:** Causal mutations identified in CD40LG gene (Reference Sequence LRG_141) of the HIGM1 patients.

Patient ID	Gene	Mutant allele	cDNA/nucleotide change	Protein Change	Mutant Type
XHIM-061A	*CD40LG*	X-linked	LRG_141t1:c.346G>T	G116C	Missense
XHIM-020A	*CD40LG*	X-linked	LRG_141t1:c.418T>G	W140G	Missense
XHIM-030A	*CD40LG*	X-linked	LRG_141t1:c.430G>A	G144R	Missense
XHIM-025A	*CD40LG*	X-linked	LRG_141t1:c.482T>A	L161Q	Missense
XHIM-050A	*CD40LG*	X-linked	LRG_141t1:c.676G>A	G226R	Missense
XHIM-029A	*CD40LG*	X-linked	LRG_141t1:c.680G>A	G227E	Missense
XHIM-049A	*CD40LG*	X-linked	LRG_141t1:c.692T>G	L231W	Missense
XHIM-037A	*CD40LG*	X-linked	**LRG_141t1:c.761C>T**	**T254M**	Missense
XHIM-058A	*CD40LG*	X-linked	**LRG_141t1:c.761C>T**	**T254M**	Missense
XHIM-047A	*CD40LG*	X-linked	LRG_141t1:c.415C>T	Q139*	Nonsense
XHIM-011A	*CD40LG*	X-linked	LRG_141t1:c.419G>A	W140*	Nonsense
XHIM-014A	*CD40LG*	X-linked	LRG_141t1:c.420G>A	W140*	Nonsense
XHIM-001A	*CD40LG*	X-linked	**LRG_141t1:c.654C>A**	C218*	Nonsense
XHIM-022A	*CD40LG*	X-linked	**LRG_141t1:c.654C>A**	C218*	Nonsense
XHIM-010A	*CD40LG*	X-linked	LRG_141t1:c.103del	Q35Rfs*2	Frameshift
XHIM-004A	*CD40LG*	X-linked	LRG_141t1:c.291_299delinsG	D97Efs*13	Frameshift
XHIM-024A	*CD40LG*	X-linked	LRG_141t1:c.511_512del	I171Lfs*29	Frameshift
XHIM-017A	*CD40LG*	X-linked	**LRG_141t1:c.158_161del**	I53Kfs*13	Frameshift
XHIM-054A	*CD40LG*	X-linked	**LRG_141t1:c.158_161del**	I53Kfs*13	Frameshift
XHIM-052A	*CD40LG*	X-linked	LRG_141t1:c.489del	R165Dfs*26	Frameshift
XHIM-016A	*CD40LG*	X-linked	LRG_141t1:c.599del	R200Nfs*42	Frameshift
XHIM-002A	*CD40LG*	X-linked	LRG_141t1:c.616_619del	L206Efs*35	Frameshift
XHIM-003A	*CD40LG*	X-linked	LRG_141t1:c.719_720del	N240Sfs*3	Frameshift
XHIM-019A	*CD40LG*	X-linked	LRG_141t1:c.157-2A>G		Splicing
XHIM-021A	*CD40LG*	X-linked	LRG_141t1:c.410-2A>G		Splicing
XHIM-036A	*CD40LG*	X-linked	LRG_141t1:c.289-28_302del		Splicing
XHIM-051A	*CD40LG*	X-linked	LRG_141t1:c.156+1G>A		Splicing
XHIM-053A	*CD40LG*	X-linked	LRG_141t1:c.346+2T>A		Splicing
XHIM-056A	*CD40LG*	X-linked	LRG_141t1:c.289-1G>C		Splicing
XHIM-057A	*CD40LG*	X-linked	LRG_141t1:c.347-1G>C		Splicing
XHIM-007A	*CD40LG*	X-linked	**LRG_141t1:c.289-2A>G**		Splicing
XHIM-009A	*CD40LG*	X-linked	**LRG_141t1:c.289-2A>G**		Splicing
XHIM-055A	*CD40LG*	X-linked	EX1_EX2del		Gross Deletion
XHIM-005A	*CD40LG*	X-linked	**EX1_EX5del**		Gross Deletion
XHIM-008A	*CD40LG*	X-linked	**EX1_EX5del**		Gross Deletion
XHIM-018A	*CD40LG*	X-linked	LRG_141t1:c.288+259_409+652delinsTCGT		Gross Deletion

Repeated mutations are in bold. CD40LG, CD40 ligand; HIGM1, X-linked immunodeficiency with hyper-IgM type 1. *translation termination (stop) codon.

**Figure 2:**
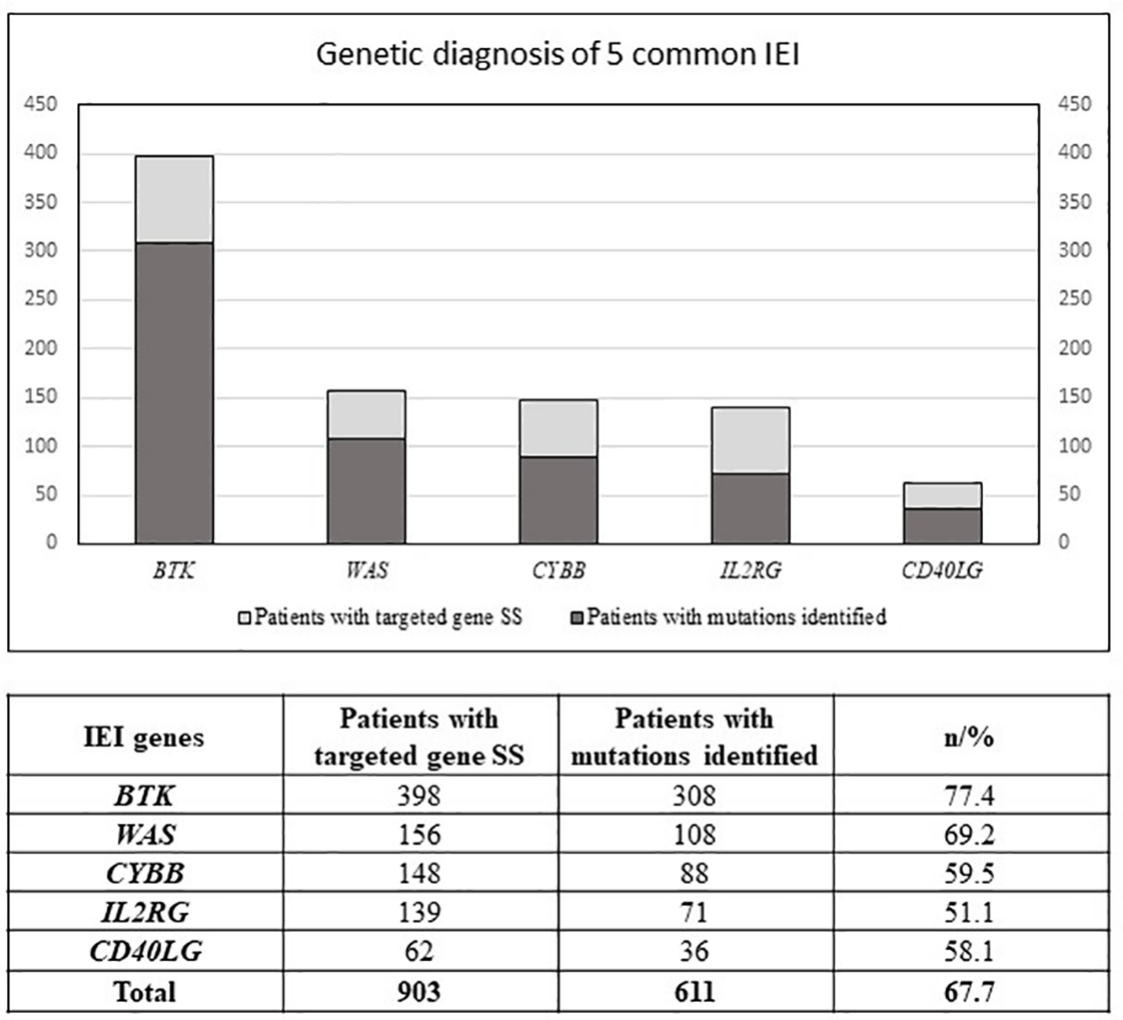
Number of patients with first round of targeted gene SS (Sanger Sequencing) performed, and number of patients with mutations identified. IEI, inborn errors of immunity; SS, Sanger sequencing; *BTK*, Bruton tyrosine kinase; *WAS*, WASP actin nucleation promoting factor; *CYBB*, cytochrome b-245 beta chain; *IL2RG*, interleukin 2 receptor subunit gamma; *CD40LG;* CD40 ligand.

**Figure 3:**
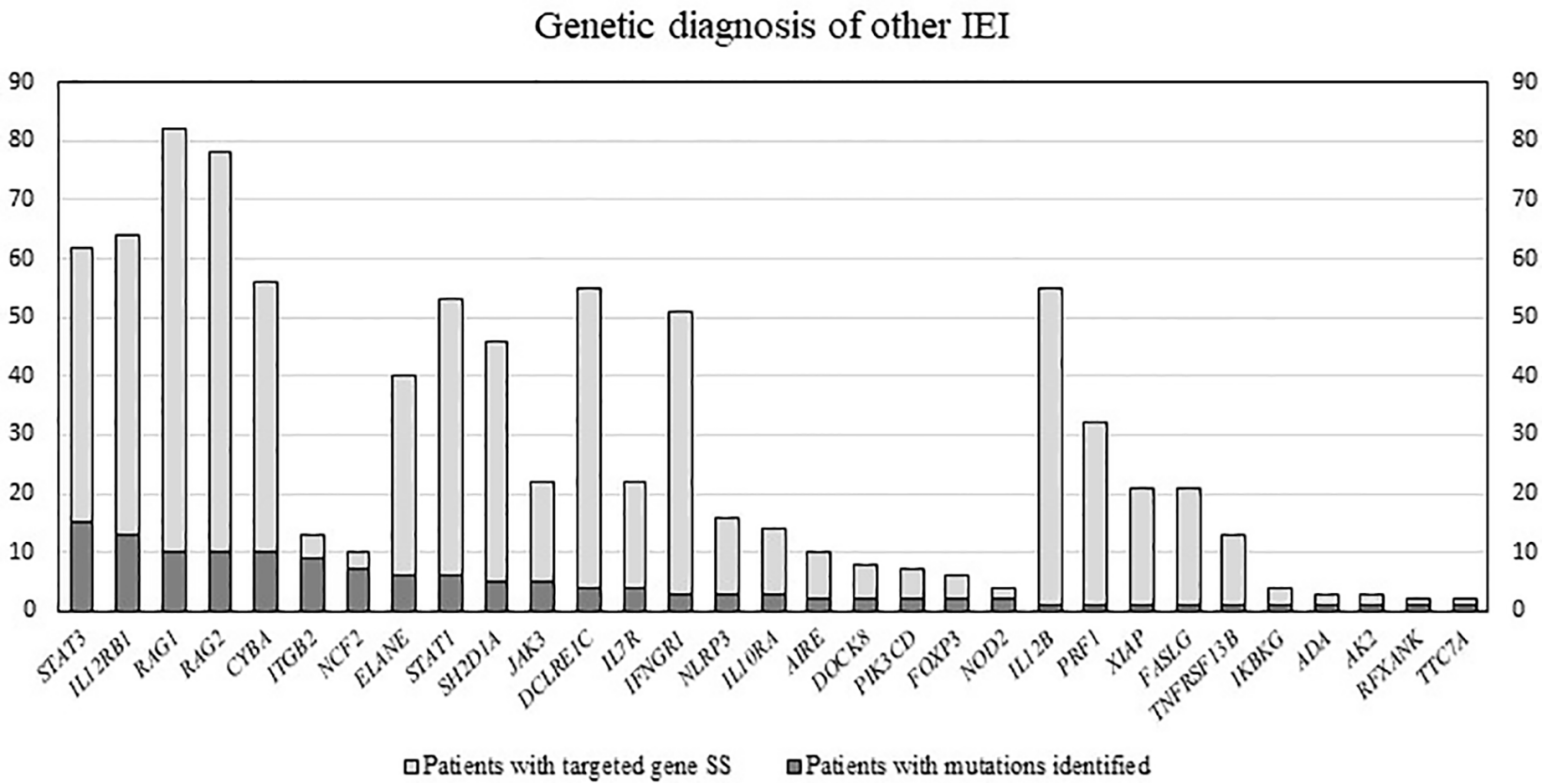
Number of patients with targeted gene SS performed, and number of patients with mutations identified. Official gene symbols approved by HGNC were used. Approved full gene names are available in HGNC. IEI, inborn errors of immunity; SS, Sanger sequencing; HGNC; HUGO Gene Nomenclature Committee.

**Figure 4:**
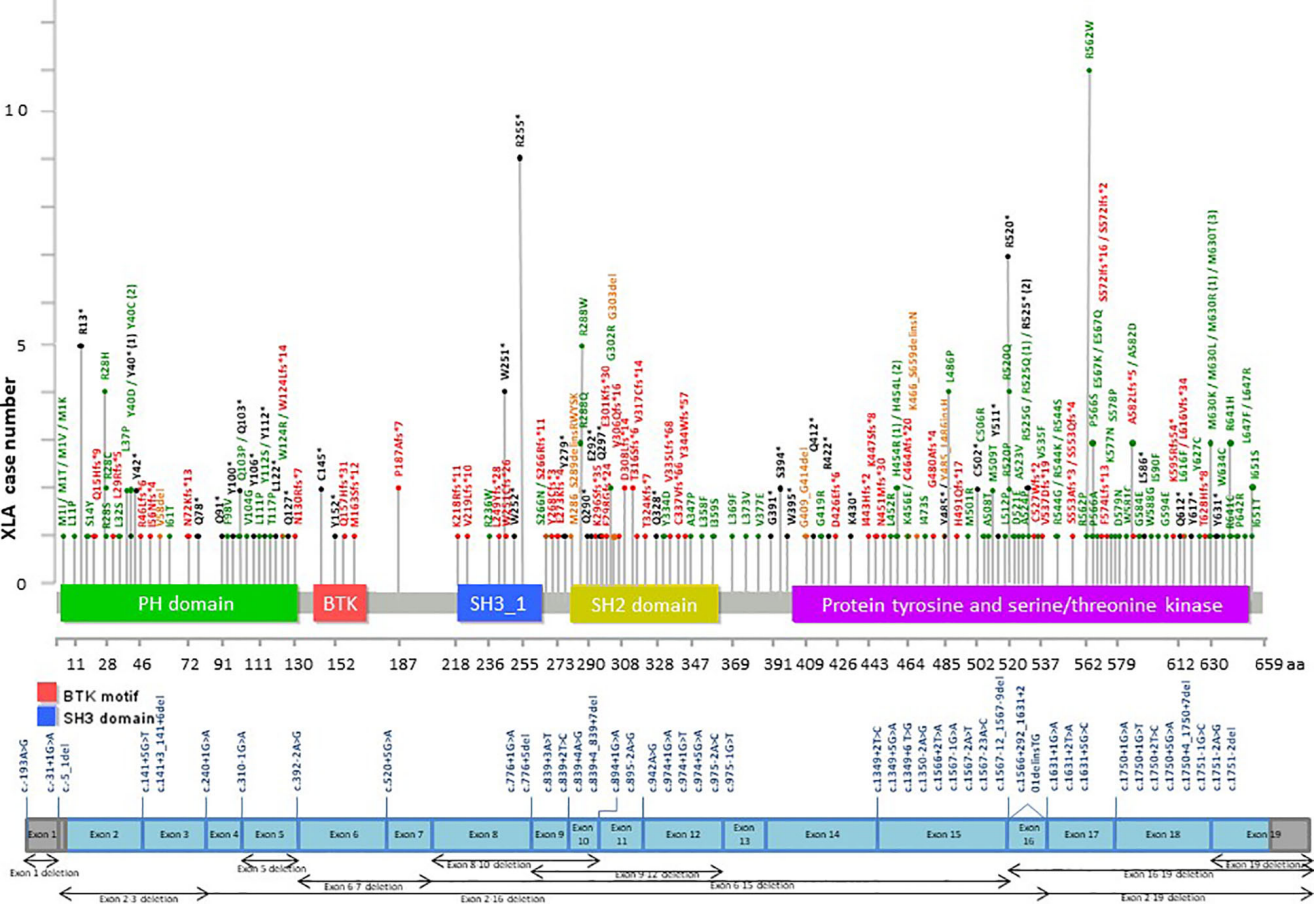
Distribution of casual mutations in various exons, exon-intron junctions and corresponding domains of *BTK* gene. The upper diagram shows the distribution and frequency of amino acid mutations in various protein domains; while the lower diagram shows the locations of splice site mutations and large deletions of the gene. *BTK*, Bruton tyrosine kinase; XLA, X-linked agammaglobulinemia; PH, Pleckstrin homology; SH2, Src homology 2; SH3. Src homology 3.

**Figure 5:**
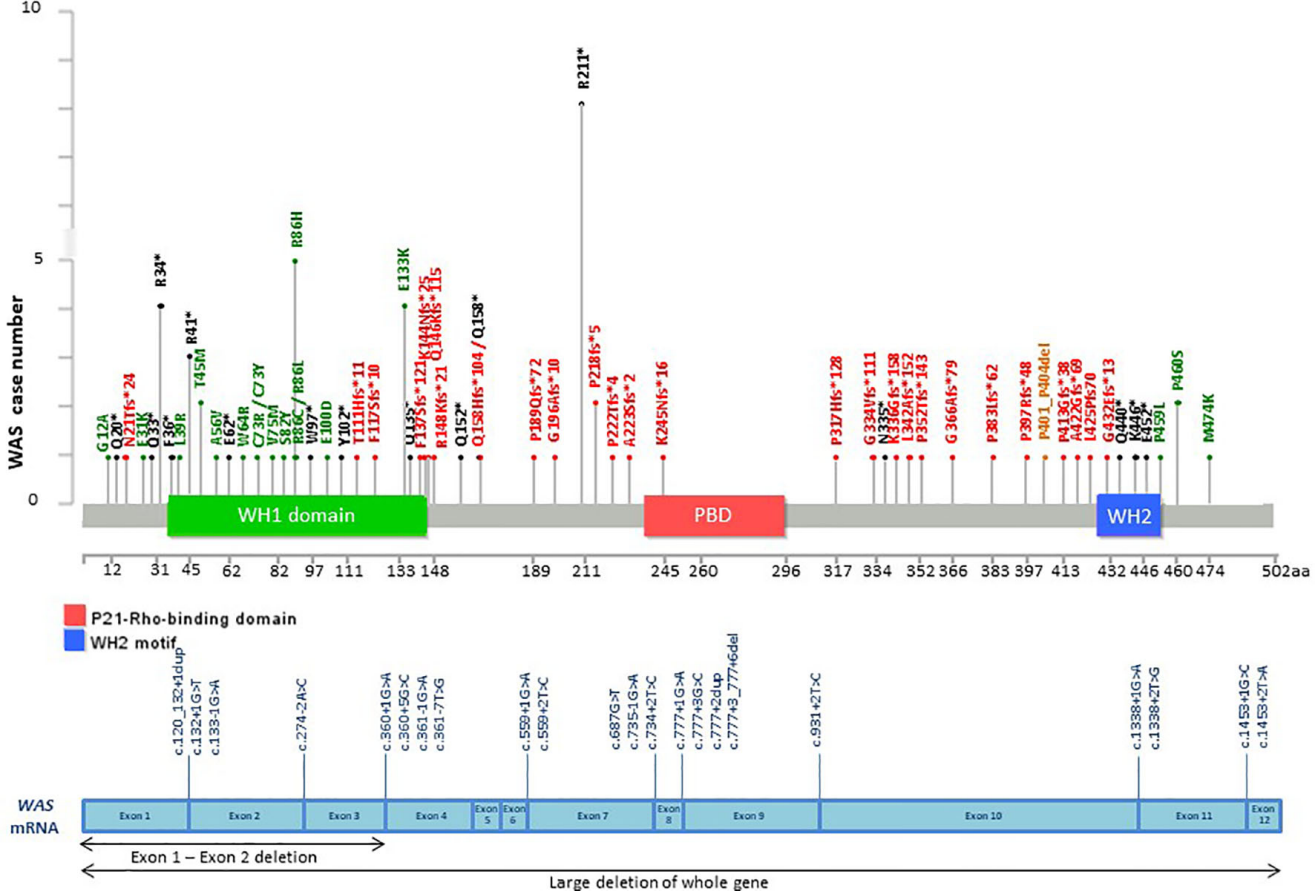
Distribution of casual mutations in various exons, exon-intron junctions and corresponding domains of *WAS* gene. The upper diagram shows the distribution and frequency of amino acid mutations in various protein domains; while the lower diagram shows the locations of splice site mutations and large deletions of the gene. *WAS*, WASP actin nucleation promoting factor; WAS, Wiskott Aldrich Syndrome; PBD, P21-Rho-binding domain; WH1, WASP homology 1 domain; WH2, WASP homology 2 domain.

**Figure 6:**
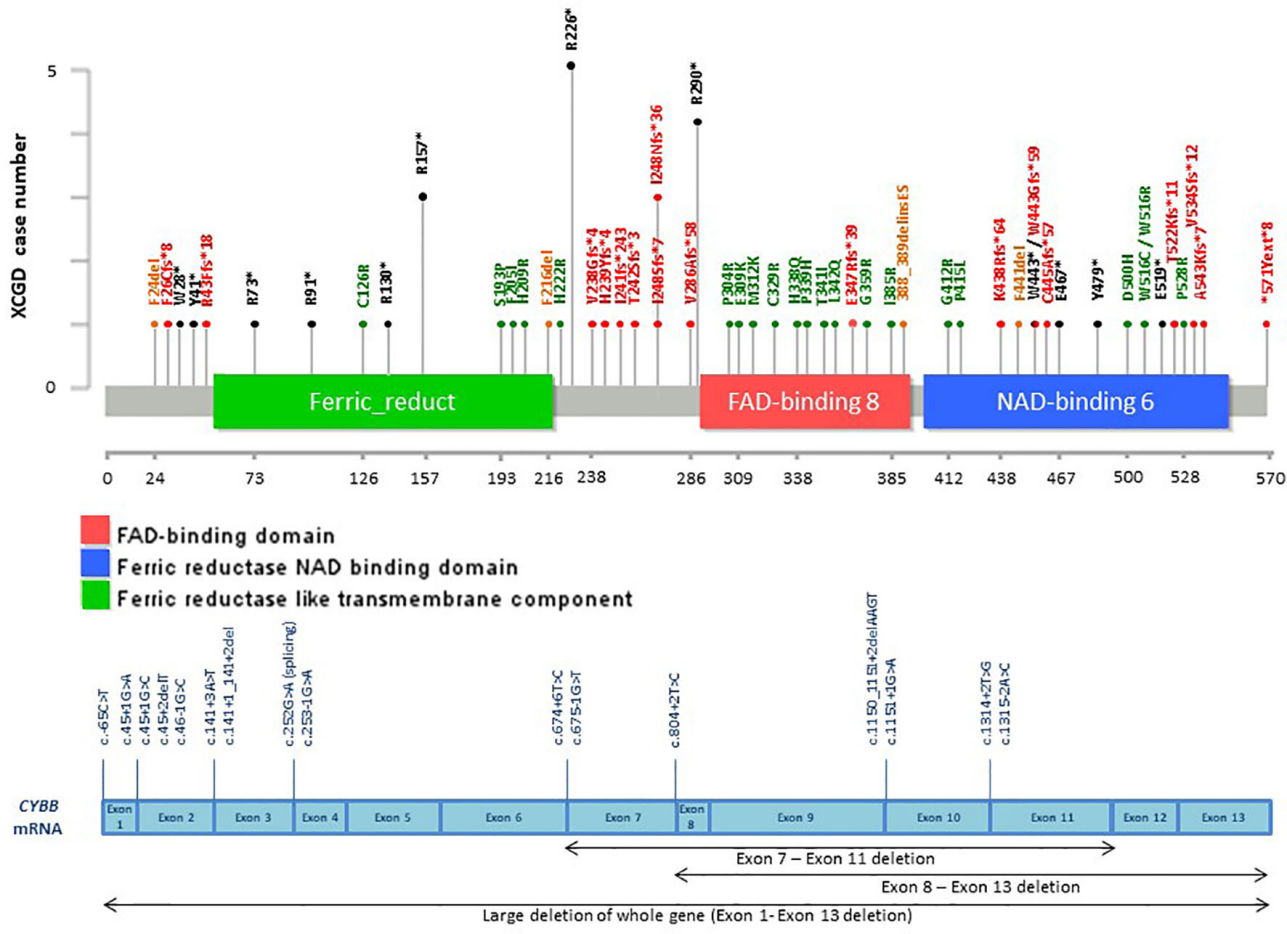
Distribution of casual mutations in various exons, exon-intron junctions and corresponding domains of *CYBB* gene. The upper diagram shows the distribution and frequency of amino acid mutations in various protein domains; while the lower diagram shows the locations of splice site mutations and large deletions of the gene. *CYBB*, cytochrome b-245 beta chain; XCGD, X-linked chronic granulomatous disease.

**Figure 7:**
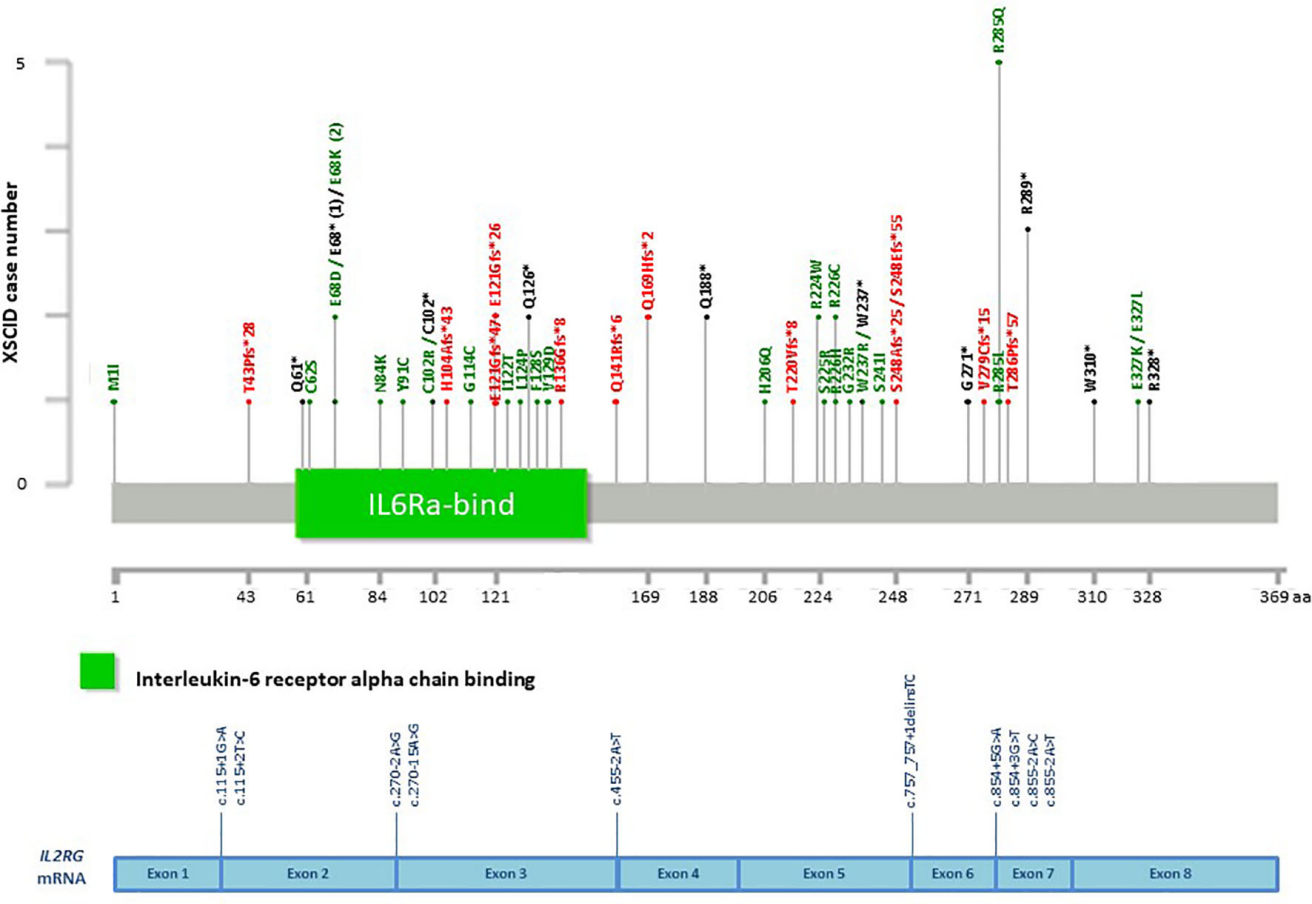
Distribution of casual mutations in various exons, exon-intron junctions and corresponding domains of *IL2RG* gene. The upper diagram shows the distribution and frequency of amino acid mutations in various protein domains; while the lower diagram shows the locations of splice site mutations and large deletions of the gene. *IL2RG*, interleukin 2 receptor subunit gamma; XSCID, X-linked severe combined immunodeficiency.

**Figure 8:**
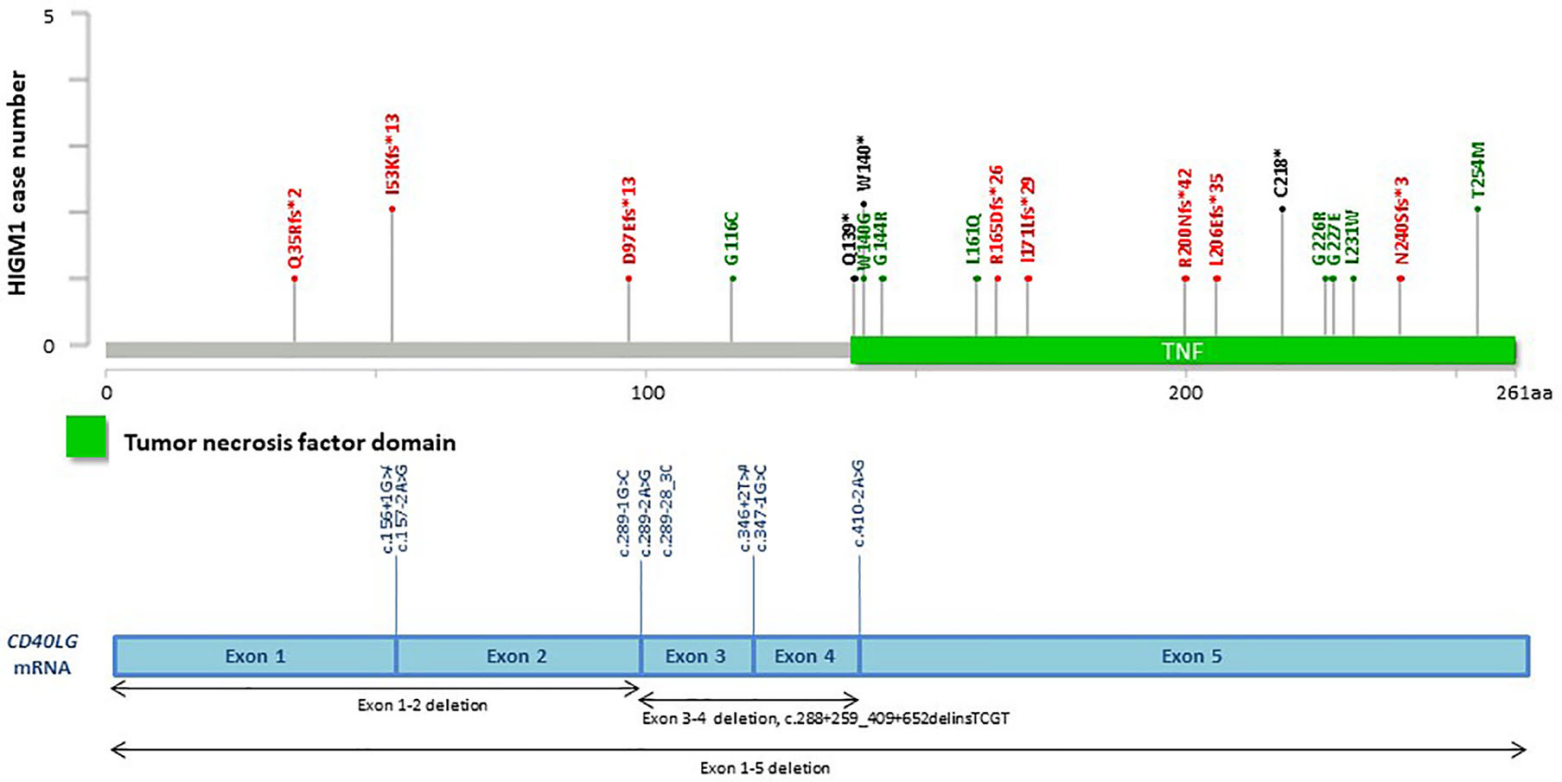
Distribution of casual mutations in various exons, exon-intron junctions and corresponding domains of *IL2RG* gene. The upper diagram shows the distribution and frequency of amino acid mutations in various protein domains; while the lower diagram shows the locations of splice site mutations and large deletions of the gene. *CD40LG;* CD40 ligand; HIGM1, X-linked immunodeficiency with hyper-IgM type 1.

Among the patients with the 5 common IEI referred, 903 single targeted gene SS were performed in the first round of screening with 611 causal mutations identified (67.7%), with the positive diagnostic rate ranging from 51.1% (*IL2RG* gene mutations for XSCID) to 77.4% (*BTK* gene mutations for XLA) (**Figure 2**). XLA is the most common referred IEI with the highest positive diagnostic rate. For the other typical and atypical IEI patients (including those with negative finding after screening for the 5 common IEI), a total of 1,121 targeted gene SS (single or multiple rounds of SS may have been done for each patient) were performed with causal mutations identified in 133 (11.9%; **Table 5** and **Figure 3**). Among the 5 common IEI, the locations of causal mutations were shown in **Figures 4**–**8**. The mutations identified include missense, nonsense, frameshift, and splicing variants. In addition, uncommon mutations such as gross deletion, in-frame deletion/insertion, start loss, stop loss and regulatory variants were identified.

**Table 5:** Number of patients with targeted gene SS performed, and number of patients with mutations identified.

IEI genes	Patients with targeted gene SS	Patients with mutations identified	%
***NCF2***	10	7	70.0
***ITGB2***	13	9	69.2
***NOD2***	4	2	50.0
***RFXANK***	2	1	50.0
***TTC7A***	2	1	50.0
***FOXP3***	6	2	33.3
***ADA***	3	1	33.3
***AK2***	3	1	33.3
***PIK3CD***	7	2	28.6
***DOCK8***	8	2	25.0
***IKBKG***	4	1	25.0
***STAT3***	62	15	24.2
***JAK3***	22	5	22.7
***IL10RA***	14	3	21.4
***IL12RB1***	64	13	20.3
***AIRE***	10	2	20.0
***NLRP3***	16	3	18.8
***IL7R***	22	4	18.2
***CYBA***	56	10	17.9
***ELANE***	40	6	15.0
***RAG2***	78	10	12.8
***RAG1***	82	10	12.2
***STAT1***	53	6	11.3
***SH2D1A***	46	5	10.9
***TNFRSF13B***	13	1	7.7
***DCLRE1C***	55	4	7.3
***IFNGR1***	51	3	5.9
***XIAP***	21	1	4.8
***FASLG***	21	1	4.8
***PRF1***	32	1	3.1
***IL12B***	55	1	1.8
***FAS***	20	0	0.0
***UNC13D***	16	0	0.0
***ICOS***	16	0	0.0
***AICDA***	13	0	0.0
***CASP10***	12	0	0.0
***MVK***	10	0	0.0
***CD40***	10	0	0.0
***UNG***	10	0	0.0
***IL10RB***	9	0	0.0
***RAB27A***	9	0	0.0
***NLRP12***	7	0	0.0
***CD79A***	7	0	0.0
***HAX1***	7	0	0.0
***TNFRSF1A***	7	0	0.0
***TYK2***	7	0	0.0
***LIG4***	6	0	0.0
***CARD9***	6	0	0.0
***RASGRP1***	6	0	0.0
***ZAP70***	6	0	0.0
***IL10***	5	0	0.0
***IL24***	5	0	0.0
***IRAK4***	5	0	0.0
***CD19***	4	0	0.0
***NCF4***	4	0	0.0
***PNP***	3	0	0.0
***IFNGR2***	3	0	0.0
***CLEC7A***	3	0	0.0
***MYD88***	3	0	0.0
***PRKCD***	3	0	0.0
***MAGT1***	2	0	0.0
***IL12A***	2	0	0.0
***ITK***	2	0	0.0
***STAT5B***	2	0	0.0
***STK4***	2	0	0.0
***TCF3***	1	0	0.0
***IL2RA***	1	0	0.0
***CXCR4***	1	0	0.0
***LRBA***	1	0	0.0
***TCIRG1***	1	0	0.0
***CLCN7***	1	0	0.0
***FERMT3***	1	0	0.0
***GATA2***	1	0	0.0
***IL1RN***	1	0	0.0
***IL36RN***	1	0	0.0
***IRF8***	1	0	0.0
***LAT***	1	0	0.0
***PGM3***	1	0	0.0
***PSMB8***	1	0	0.0
**Total**	**1121**	**133**	**11.9**

Official gene symbols approved by HGNC were used. Approved full gene names are available in HGNC. IEI, inborn errors of immunity; SS, Sanger sequencing; HGNC, HUGO Gene Nomenclature Committee. Sum of patients are in bold.

## Discussions

Using one single round of targeted gene SS in our study was successful in diagnosing 611 of the 903 patients (67.7%) suspected to have one of the 5 common IEI, i.e., XLA (77.4%), WAS (69.2%), XCGD (59.5%), XHIM (58.1%), and XSCID (51.1%), definitively. These 5 IEI are X-linked which renders the genetic diagnosis more readily and accurately achieved. At the clinical level, a positive family history of maternal uncles or male cousins affected with similar clinical and immunological phenotypes, suggestive of X-linked pattern of inheritance, will be the first clue. Moreover, the clinical and immunological phenotypes of these 5 IEI are relatively uniform, except for XSCID, which could have multiple phenotypes due to hypomorphic mutations of *IL2RG* gene as well as presence of multiple genes giving rise to similar immunological phenotypes. The immunophenotype of these 5 IEI is more easily defined by laboratory tests which are less technically demanding and more available, such as complete blood count, lymphocyte subsets, immunoglobulin profile, and the nitroblue tetrazolium test (6). Though the diagnostic resources and experience of referring clinicians could differ among different centres, affecting the accuracy of the diagnosis for these 5 IEI, our findings demonstrated that the individual positive diagnostic rate is much higher than that for the other IEI (11.9%), see **Supplementary Figure 1**. In addition, referring clinicians can learn from our e-consultation and diagnostic algorithm to further improve the diagnostic rate. More importantly, these 5 IEI occur at much higher rates than the rest of the 400 IEI, resulting in a higher level of awareness among paediatricians, hence earlier recognition, and referral for definitive genetic diagnosis than the less common IEI.

At the genetic diagnostic level, X-linked IEI is easier to diagnose than autosomal recessive IEI in non-consanguineous population, because identification of causal mutation in a single allele is sufficient. Moreover, there is no pitfall of missing the identification of heterozygous gross deletion by Sanger sequencing as in autosomal IEI with PCR still positive in such cases. For the X-linked genes, gross deletion will be picked up by negative PCR, and then one can confirm the deletion in each exon by multiplex PCR, co-amplification of both target and reference gene, with normal control. Due to limitation of our primers design, causal mutations within those intronic and regulatory regions may not be included in the PCR regions, and hence cannot be identified. Nevertheless, the strengths of targeted gene SS include >99% high accuracy, fast turnaround time, low cost, with fewer variants of uncertain significance and no secondary findings (3, 4). Therefore, doing one round of single specific targeted gene SS remains the first-tier genetic test for patients suspected to have one of these 5 common IEI in our laboratory.

Apart from these 5 common IEI, there were 2 more IEI with over 50% genetic diagnostic success rates in our study using targeted gene SS, i.e., leucocyte adhesion deficiency type 1 (LAD1) and autosomal recessive chronic granulomatous disease (AR-CGD) due to neutrophil cytosolic factor2 (*NCF2*) gene mutations. For LAD1, the clinical and immunological phenotype is uniform with little variation, and LAD1 occurs at a much higher frequency than the other two types of LAD. With flow cytometric analysis of CD18, followed by integrin subunit beta 2 (*ITGB2*) gene SS, LAD1 can be diagnosed easily (46). Our one round of single targeted gene SS was successful in diagnosing 9 of the 13 patients (69.2%) suspected to have LAD1. As for AR-CGD due to *NCF2*gene mutations, the success rate of targeted gene SS in making the genetic diagnosis was 70% in our study (7 out of 10 patients), but this was achieved by doing multiple AR-CGD genes at the same time, after failing to identify the genetic mutation for *CYBB* gene in male patients suspected to have CGD. Therefore, the 70% success rate was not after doing just one round of single targeted gene SS, but after multiple rounds of targeted gene SS of genes responsible for AR-CGD.

For the rest of the IEI, the success rates of achieving genetic diagnosis for each of these IEI after targeted gene SS were mostly under one-third, and in most cases, we had to do multiple rounds of targeted gene SS, with an overall success rate of only 10.9%. Therefore, whole exome sequencing (WES) is now our preferred first-tier genomic test for all the IEI except the 5 most common X-linked IEI and LAD1. However, exceptions do occur, such as AR-CGD due to *NCF1* gene, which has pseudogenes, rendering both SS and WES not able to identify the causal mutations due to poor and limited coverage of sequences shared with pseudogenes. Fortunately, 97% of affected alleles in patients previously reported with p47-phox deficiency carry a hot spot mutation of “GT”deletion (ΔGT) in exon 2 of neutrophil cytosolic factor 1 (*NCF1*) gene (47). One can therefore simply identify the hot spot mutation by GeneScan^®^ analysis as shown in **Supplementary Figure 2** before proceeding to sequencing of the coding region. This approach was adopted by us to save time and cost

All in all, we were able to diagnose 744 of the 1376 patients (54.1%) referred to us suspected to have IEI, using targeted genes SS, with an average of 1.47 such tests per patient (ranging from 1 to 10). However, 632 of these 1376 patients (45.9%) of the referred patients remained genetically undiagnosed after single or multiple rounds of targeted gene SS.

With the availability of WES in 2009, we deployed this technology for selected undiagnosed IEI patients. Our first WES case for a male infant with early-onset inflammatory bowel disease (IBD) in 2009 resulted in the discovery of interleukin 10 receptor subunit alpha (*IL10RA*) gene mutations as the underlying cause of early-onset IBD (27), at about the same time when aberrant interleukin 10 (IL10) pathway was implicated as the underlying cause for early-onset IBD by another group using linkage analysis (48). Since then, we have incorporated WES more readily into our diagnostic algorithm, because of the cost coming down as well as developing our own in-house bioinformatic tools and analysis, resulting in discovery of novel IEI (49, 50). We shall review in future our experience in using WES for patients with suspected IEI who remain undiagnosed genetically after targeted gene SS. Comparison between targeted gene SS and NGS (whole exome sequencing WES) in our institutional service has been shown in **Supplementary Figure 3**. In general, WES will have wider applications, but longer turnover time compared with SS under the service provided by our centre. However, if both the financial and human resource (laboratory staffs and bioinformaticians) is not a limiting factor, rapid WES may be considered to set up for those urgent cases with immediate clinical management decision (51).

In conclusion, single targeted gene SS should remain the first-tier genetic test for patients suspected to have one of the 5 common X-linked IEI before offering genomic tests such as WES or targeted gene panel (52). Flow chart of our current diagnostic algorithm, with the description of progressive changes in our bioinformatic analysis, has been provided as reference (**Supplementary Figure 4**). We propose IEI centres in less resourced Asian and African countries and regions could consider setting up targeted gene SS for these 6 IEI which would yield a high enough success rate of genetic diagnosis in a significant number of IEI patients to become cost-effective (6, 53).

## Data Availability Statement

The original contributions presented in the study are included in the article/**Supplementary Material** Further inquiries can be directed to the corresponding author.

## Ethics Statement

The studies involving human participants were reviewed and approved by Clinical Research Ethics Review Board of The University of Hong Kong and Queen Mary Hospital (Ref. no. UW 08-301). Written informed consent to participate in this study was provided by the participants’ legal guardian/next of kin.

## Author Contributions

Y-LL conceptualized the study. YL, XY, WT, PL, WY, and DL designed the study. K-WC, C-YW, SF, and PM performed genetic study. K-WC, and DL curated mutations. PL and DL phenotyped the patients. K-WC, C-YW, XY, and DL analyzed data. K-WC and C-YW drafted the manuscript. Other authors referred patients and provided clinical care and clinical data. All authors critically reviewed the manuscript. All authors contributed to the article and approved the submitted version.

## Funding

This work was supported by the Hong Kong Society for Relief of Disabled Children and Jeffrey Modell Foundation.

## Conflict of Interest

The authors declare that the research was conducted in the absence of any commercial or financial relationships that could be construed as a potential conflict of interest.

## Publisher’s Note

All claims expressed in this article are solely those of the authors and do not necessarily represent those of their affiliated organizations, or those of the publisher, the editors and the reviewers. Any product that may be evaluated in this article, or claim that may be made by its manufacturer, is not guaranteed or endorsed by the publisher.
