# Supplementary material for: Targeted Gene Sanger Sequencing Should Remain the First-Tier Genetic Test for Children Suspected to Have the Five Common X-Linked Inborn Errors of Immunity
Source: Front Immunol. 2022 Jul 8;13:883446. doi: 10.3389/fimmu.2022.883446 (PMC9304939; doi:10.3389/fimmu.2022.883446)
Supplement: Supplementary file 1 [file Presentation_1.pptx]

## Slide 1
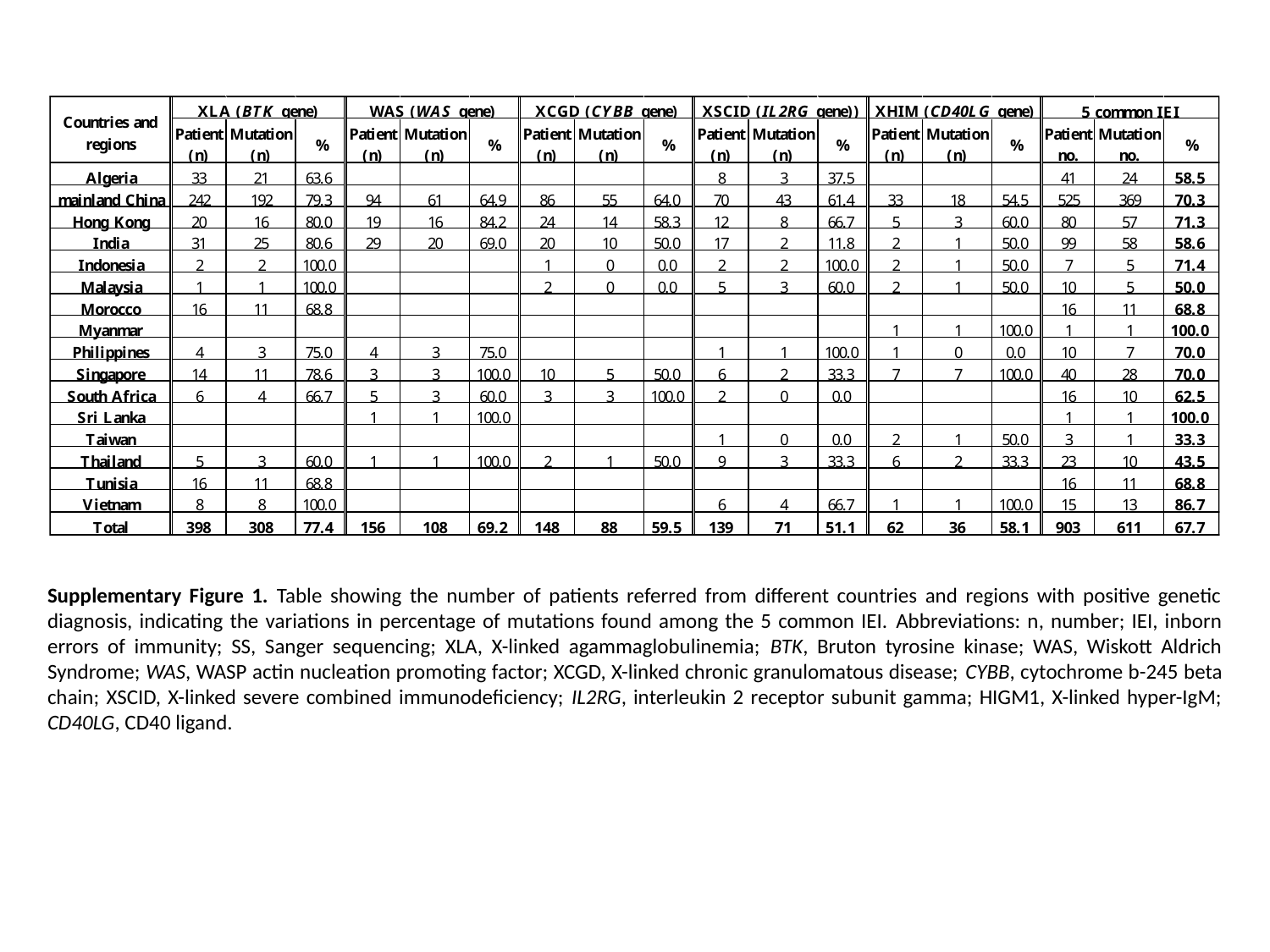

Supplementary Figure 1. Table showing the number of patients referred from different countries and regions with positive genetic diagnosis, indicating the variations in percentage of mutations found among the 5 common IEI. Abbreviations: n, number; IEI, inborn errors of immunity; SS, Sanger sequencing; XLA, X-linked agammaglobulinemia; BTK, Bruton tyrosine kinase; WAS, Wiskott Aldrich Syndrome; WAS, WASP actin nucleation promoting factor; XCGD, X-linked chronic granulomatous disease; CYBB, cytochrome b-245 beta chain; XSCID, X-linked severe combined immunodeficiency; IL2RG, interleukin 2 receptor subunit gamma; HIGM1, X-linked hyper-IgM; CD40LG, CD40 ligand.
