# Supplementary material for: Targeted Gene Sanger Sequencing Should Remain the First-Tier Genetic Test for Children Suspected to Have the Five Common X-Linked Inborn Errors of Immunity
Source: Front Immunol. 2022 Jul 8;13:883446. doi: 10.3389/fimmu.2022.883446 (PMC9304939; doi:10.3389/fimmu.2022.883446)
Supplement: Supplementary file 2 [file Presentation_2.pptx]

## Slide 1
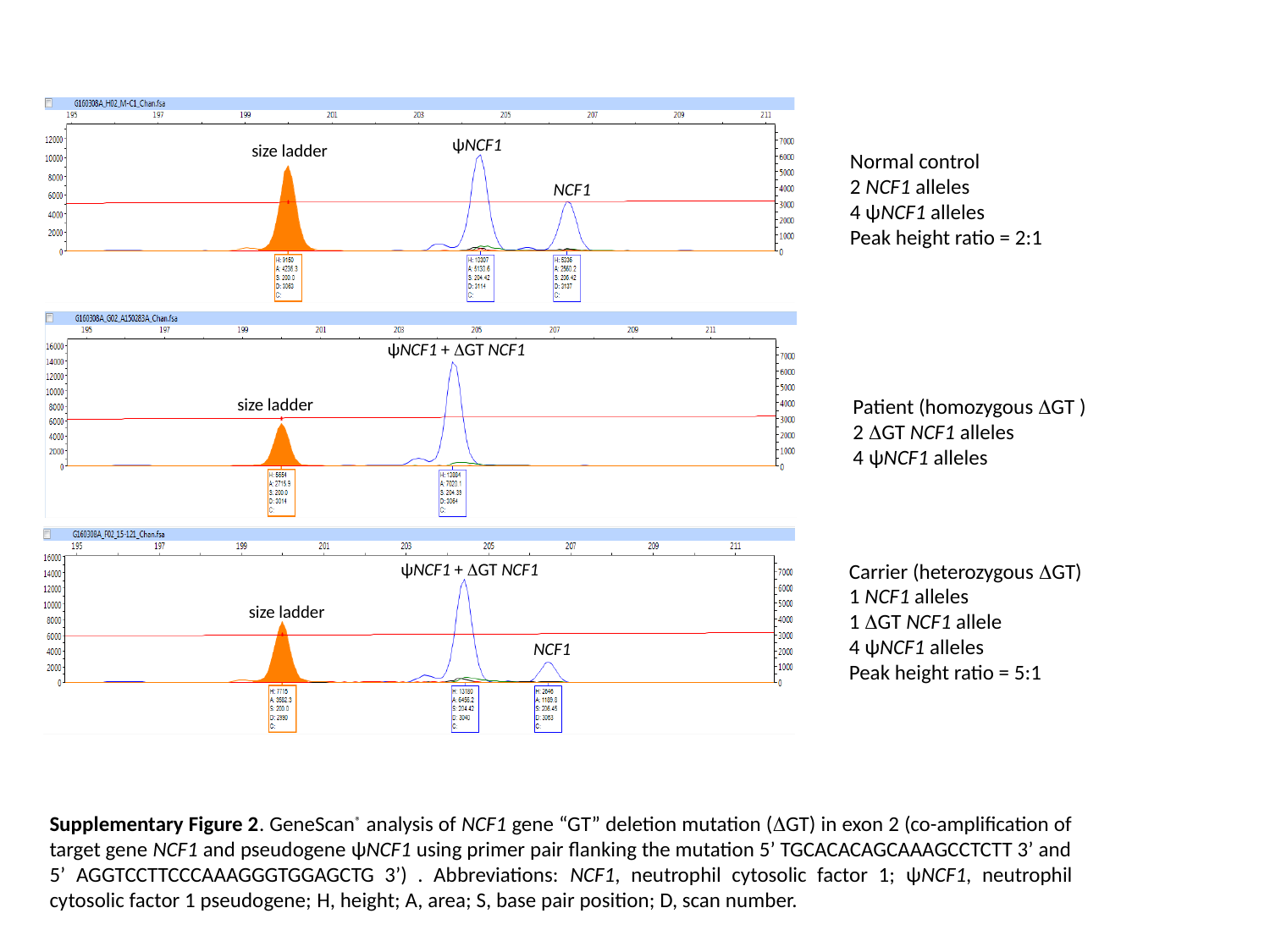

ψNCF1
size ladder
Normal control
2 NCF1 alleles
4 ψNCF1 alleles
Peak height ratio = 2:1
NCF1
ψNCF1 + ΔGT NCF1
size ladder
Patient (homozygous ΔGT )
2 ΔGT NCF1 alleles
4 ψNCF1 alleles
ψNCF1 + ΔGT NCF1
Carrier (heterozygous ΔGT)
1 NCF1 alleles
1 ΔGT NCF1 allele
4 ψNCF1 alleles
Peak height ratio = 5:1
size ladder
NCF1
Supplementary Figure 2. GeneScan® analysis of NCF1 gene “GT” deletion mutation (ΔGT) in exon 2 (co-amplification of target gene NCF1 and pseudogene ψNCF1 using primer pair flanking the mutation 5’ TGCACACAGCAAAGCCTCTT 3’ and 5’ AGGTCCTTCCCAAAGGGTGGAGCTG 3’) . Abbreviations: NCF1, neutrophil cytosolic factor 1; ψNCF1, neutrophil cytosolic factor 1 pseudogene; H, height; A, area; S, base pair position; D, scan number.
