# Supplementary material for: Targeted Gene Sanger Sequencing Should Remain the First-Tier Genetic Test for Children Suspected to Have the Five Common X-Linked Inborn Errors of Immunity
Source: Front Immunol. 2022 Jul 8;13:883446. doi: 10.3389/fimmu.2022.883446 (PMC9304939; doi:10.3389/fimmu.2022.883446)
Supplement: Supplementary file 3 [file Presentation_3.pptx]

## Slide 1
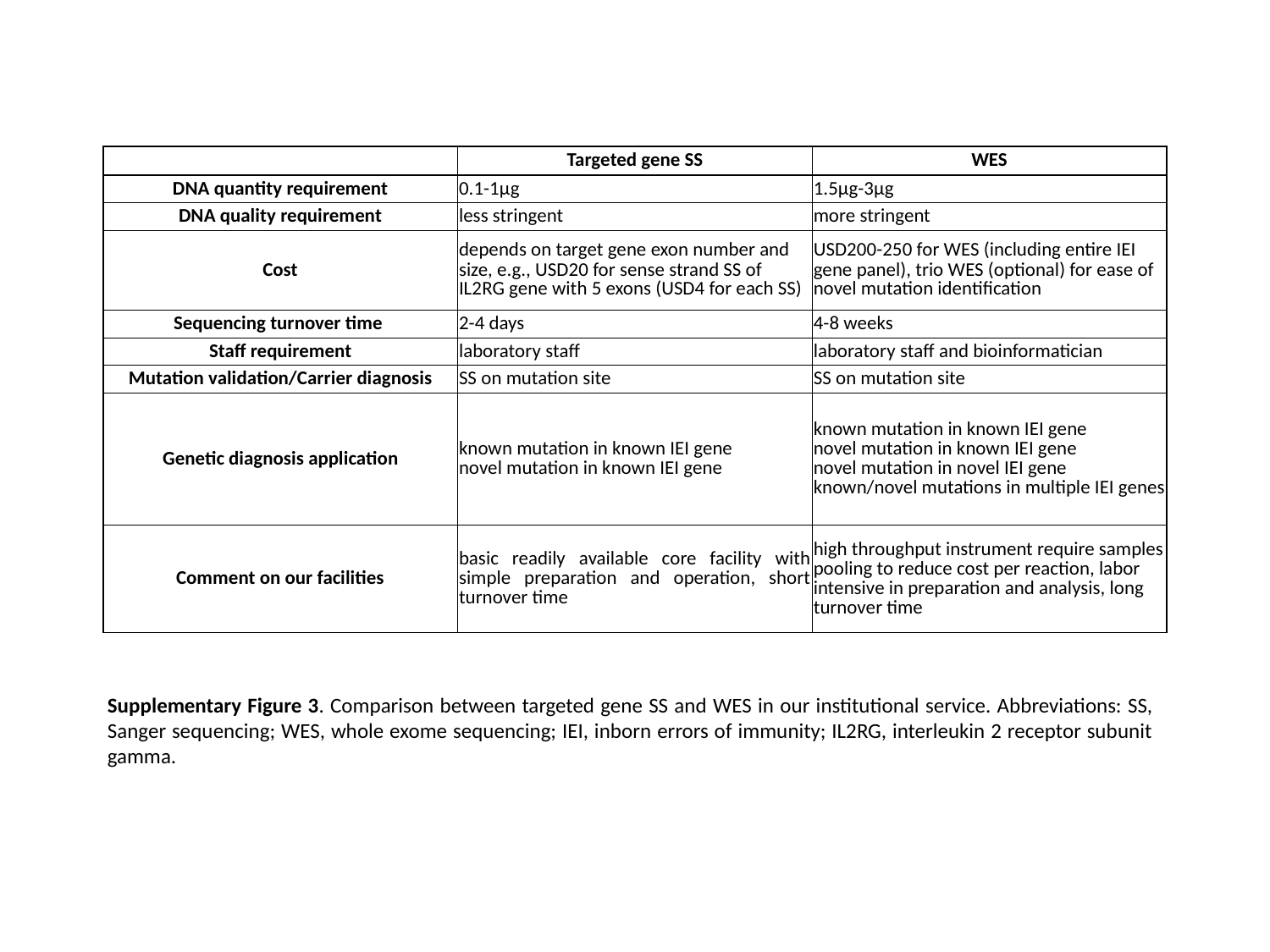

| | Targeted gene SS | WES |
| --- | --- | --- |
| DNA quantity requirement | 0.1-1µg | 1.5µg-3µg |
| DNA quality requirement | less stringent | more stringent |
| Cost | depends on target gene exon number and size, e.g., USD20 for sense strand SS of IL2RG gene with 5 exons (USD4 for each SS) | USD200-250 for WES (including entire IEI gene panel), trio WES (optional) for ease of novel mutation identification |
| Sequencing turnover time | 2-4 days | 4-8 weeks |
| Staff requirement | laboratory staff | laboratory staff and bioinformatician |
| Mutation validation/Carrier diagnosis | SS on mutation site | SS on mutation site |
| Genetic diagnosis application | known mutation in known IEI gene novel mutation in known IEI gene | known mutation in known IEI gene novel mutation in known IEI gene novel mutation in novel IEI gene known/novel mutations in multiple IEI genes |
| Comment on our facilities | basic readily available core facility with simple preparation and operation, short turnover time | high throughput instrument require samples pooling to reduce cost per reaction, labor intensive in preparation and analysis, long turnover time |
Supplementary Figure 3. Comparison between targeted gene SS and WES in our institutional service. Abbreviations: SS, Sanger sequencing; WES, whole exome sequencing; IEI, inborn errors of immunity; IL2RG, interleukin 2 receptor subunit gamma.
