# Supplementary material for: Targeted Gene Sanger Sequencing Should Remain the First-Tier Genetic Test for Children Suspected to Have the Five Common X-Linked Inborn Errors of Immunity
Source: Front Immunol. 2022 Jul 8;13:883446. doi: 10.3389/fimmu.2022.883446 (PMC9304939; doi:10.3389/fimmu.2022.883446)
Supplement: Supplementary file 4 [file Presentation_4.pptx]

## Slide 1
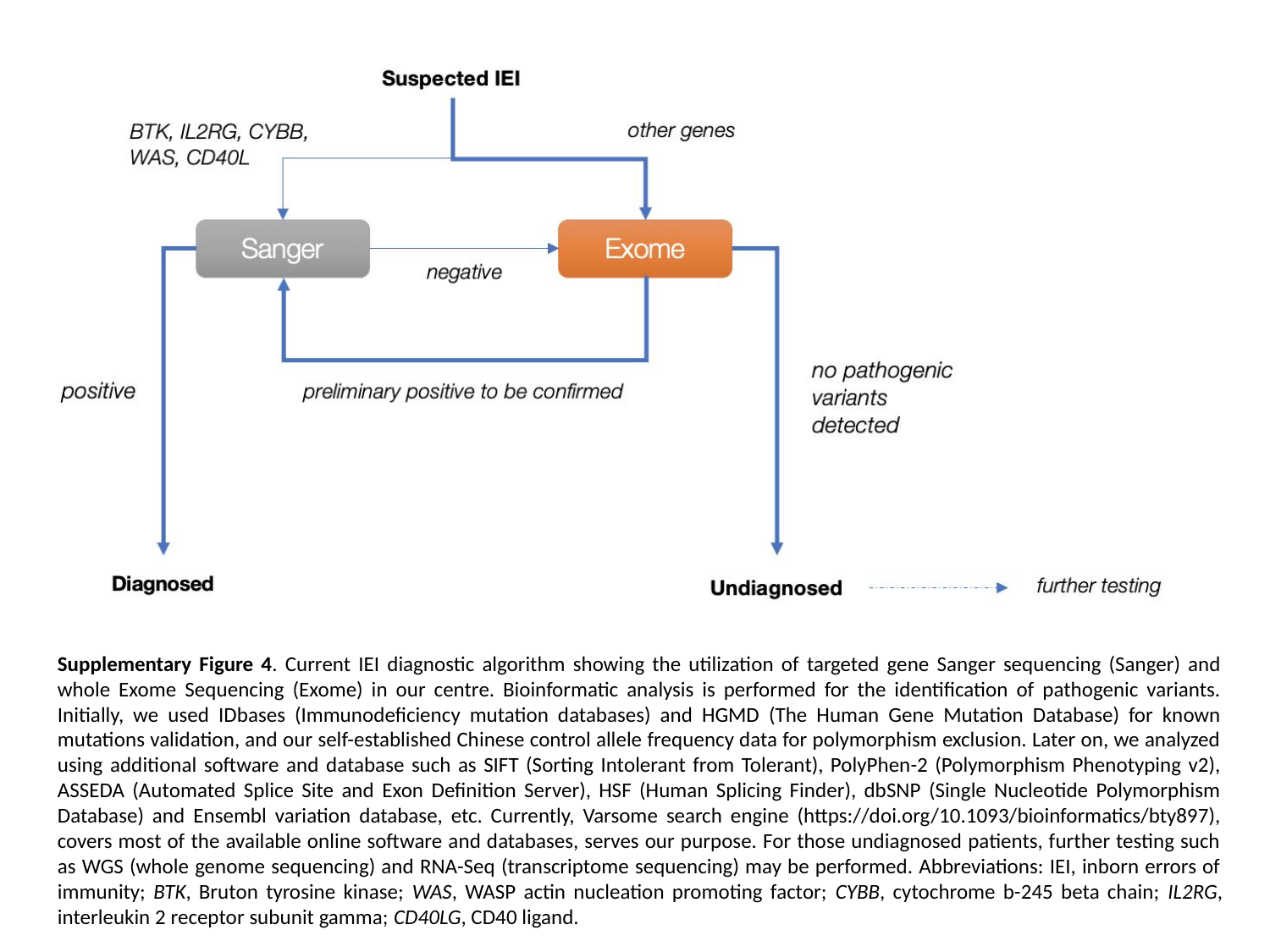

Supplementary Figure 4. Current IEI diagnostic algorithm showing the utilization of targeted gene Sanger sequencing (Sanger) and whole Exome Sequencing (Exome) in our centre. Bioinformatic analysis is performed for the identification of pathogenic variants. Initially, we used IDbases (Immunodeficiency mutation databases) and HGMD (The Human Gene Mutation Database) for known mutations validation, and our self-established Chinese control allele frequency data for polymorphism exclusion. Later on, we analyzed using additional software and database such as SIFT (Sorting Intolerant from Tolerant), PolyPhen-2 (Polymorphism Phenotyping v2), ASSEDA (Automated Splice Site and Exon Definition Server), HSF (Human Splicing Finder), dbSNP (Single Nucleotide Polymorphism Database) and Ensembl variation database, etc. Currently, Varsome search engine (https://doi.org/10.1093/bioinformatics/bty897), covers most of the available online software and databases, serves our purpose. For those undiagnosed patients, further testing such as WGS (whole genome sequencing) and RNA-Seq (transcriptome sequencing) may be performed. Abbreviations: IEI, inborn errors of immunity; BTK, Bruton tyrosine kinase; WAS, WASP actin nucleation promoting factor; CYBB, cytochrome b-245 beta chain; IL2RG, interleukin 2 receptor subunit gamma; CD40LG, CD40 ligand.
